# Altered distribution, aggregation, and protease resistance of cellular prion protein following intracranial inoculation

**DOI:** 10.1371/journal.pone.0219457

**Published:** 2019-07-10

**Authors:** Anne Ward, Jason R. Hollister, Young Pyo Choi, Brent Race, Katie Williams, Daniel W. Shoup, Roger A. Moore, Suzette A. Priola

**Affiliations:** Laboratory of Persistent Viral Diseases, Rocky Mountain Laboratories, National Institute of Allergy and Infectious Diseases, National Institutes of Health, Hamilton, Montana, United States of America; Creighton University, UNITED STATES

## Abstract

Prion protein (PrP^C^) is a protease-sensitive and soluble cell surface glycoprotein expressed in almost all mammalian cell types. PrP^Sc^, a protease-resistant and insoluble form of PrP^C^, is the causative agent of prion diseases, fatal and transmissible neurogenerative diseases of mammals. Prion infection is initiated via either ingestion or inoculation of PrP^Sc^ or when host PrP^C^ stochastically refolds into PrP^Sc^. In either instance, the early events that occur during prion infection remain poorly understood. We have used transgenic mice expressing mouse PrP^C^ tagged with a unique antibody epitope to monitor the response of host PrP^C^ to prion inoculation. Following intracranial inoculation of either prion-infected or uninfected brain homogenate, we show that host PrP^C^ can accumulate both intra-axonally and within the myelin membrane of axons suggesting that it may play a role in axonal loss following brain injury. Moreover, in response to the inoculation host PrP^C^ exhibits an increased insolubility and protease resistance similar to that of PrP^Sc^, even in the absence of infectious prions. Thus, our results raise the possibility that damage to the brain may be one trigger by which PrP^C^ stochastically refolds into pathogenic PrP^Sc^ leading to productive prion infection.

## Introduction

Prion diseases are rare, transmissible and inevitably fatal neurodegenerative diseases characterized by the accumulation of an infectious, aggregated and insoluble protease-resistant isoform (PrP^Sc^) of the normally soluble and protease-sensitive host prion protein (PrP^C^). They have been identified in multiple mammalian species including sheep (scrapie), deer (chronic wasting disease), cattle (bovine spongiform encephalopathy) and humans (Creutzfeldt-Jakob disease). Prion diseases are distinguished neuropathologically by significant gliosis and cellular loss in the brain which leads to the spongiform change that is a hallmark of these diseases. The causative agent of prion diseases, known as the prion, is composed primarily of PrP^Sc^. Prions replicate via a mechanism known as seeded polymerization whereby aggregates of PrP^Sc^ bind to host PrP^C^ and induce its conformational conversion into new PrP^Sc^ [1, 2]. Over a period of months to years, persistent prion replication leads to the accumulation of PrP^Sc^ in the brain and eventually clinical prion disease. Interestingly, prion diseases can be initiated either via exposure to exogenous prions or by the spontaneous refolding of the host PrP^C^ molecule into PrP^Sc^. It is unclear what may trigger host PrP^C^ to spontaneously refold into PrP^Sc^ but the end result is sporadic Creutzfeldt-Jakob disease (sCJD), the most common form of prion disease in humans [3]. Regardless of the origin of infection, there are no clinically proven preventative or therapeutic treatments for prion diseases and they are inevitably fatal.

Understanding how prions initiate infection, as well as the host response to infection, could provide new opportunities for blocking prion infection and spread. *In vitro*, the mechanisms underlying prion infection have been studied extensively using a variety of cell lines susceptible to prion infection (for review see [4]). *In vivo*, the need to distinguish the three types of PrP molecules involved in establishing prion infection (exogenous PrP^Sc^, host cell PrP^C^, and host cell-derived PrP^Sc^), as well as the rapid dissemination of prions out of the brain following intracranial inoculation [5] has made it difficult to detect exogenous PrP^Sc^ within the first few days post-infection [6, 7]. As a result, the uptake and spread of PrP^Sc^ has been studied using mice in which the prion protein gene *Prnp* has been knocked out (PrP^KO^ mice) [8–10] using either inoculation of small volumes of prions into a limited area [10, 11] or peripheral routes of inoculation [8, 11–17], and by inoculation of fluorophore [14, 17] or radiolabeled [18] PrP^Sc^. All of these studies have demonstrated that PrP^Sc^ uptake and spread is detectable within the first few hours post-infection, with concentration of the inoculum at the injection site allowing detection of exogenous PrP^Sc^ in as little as 15 minutes in the gut [9, 11] and 30 minutes in the brain [10]. Thus, consistent with *in vitro* results [4], PrP^Sc^ uptake and spread also appear to occur rapidly during early prion infection *in vivo*.

Detection of *de novo* PrP^Sc^ formed within the first few days following prion infection is hampered by difficulties in distinguishing exogenous PrP^Sc^ molecules from endogenous host prion protein. A recent study comparing PrP^Sc^ levels in the brains of prion-inoculated wild-type mice to prion-inoculated PrP^KO^ mice suggested that acute PrP^Sc^ formation could be detected 3 days after stereotactic inoculation of mouse prions into the brain [10]. Increased PrP staining in the area of the needle track was also observed that was consistent with either the presence of host PrP^C^ in axons or *de novo* host-derived PrP^Sc^ [10]. In the absence of a way to distinguish exogenous PrP^Sc^ from the PrP of the host, however, an acute response of endogenous host PrP^C^ to infection or inoculation could potentially confound the identification of newly formed PrP^Sc^ and the areas of the brain where it occurs.

In the current study, we have used transgenic mice expressing mouse prion protein with the epitope to the hamster PrP-specific monoclonal antibody 3F4 [19]. The 3F4 antibody does not recognize PrP^Sc^ from wild-type mice [20], thus allowing us to unambiguously identify host PrP during prion infection. We show that, following intracranial inoculation of brain homogenate, endogenous host PrP^C^ exhibits increased aggregation and protease resistance in both prion and normal brain homogenate-inoculated animals for at least 2 weeks following inoculation. Host PrP^C^ distribution in the areas of inoculation damage was altered from a primarily punctate, perinuclear stain to a relatively uniform staining pattern within axons. Moreover, in some degenerated axons host PrP also clearly localized within the myelin sheath along with myelin basic protein and proteolipid protein. Our results show that host PrP^C^ can acquire and maintain PrP^Sc^-like characteristics even in the absence of prions suggesting that it could be difficult to distinguish *bona fide*, newly formed PrP^Sc^ from host PrP^C^ during early prion infection. Moreover, our data demonstrate that damage to the brain results in changes to both the biochemical properties of PrP^C^ and its cellular distribution and suggest that aggregated and protease-resistant PrP^C^ may play a role in axonal damage following an insult to the brain.

## Materials and methods

### Antibodies

For fluorescent immunohistochemistry, primary antibody specificities and dilutions used are summarized in S1 Table. Secondary antibodies from ThermoFisher Scientific were diluted 1:500 and were either Alexa Fluor 488 goat anti-rabbit, streptavidin-conjugated Alexa Fluor 568, or streptavidin-conjugated Alexa Fluor 594.

For western blot analysis, the primary antibody was either the anti-hamster PrP mouse monoclonal antibody 3F4 conjugated to biotin (1:10,000 dilution), 3F4 derived in-house from hybridoma supernatant (1:1,000 dilution), or the anti-mouse PrP antibody 6D11 (1:5,000 dilution). Secondary antibodies were either streptavidin-conjugated horse radish peroxidase (HRP, Cell Signaling Technologies) diluted 1:250,000, or sheep anti-mouse IgG conjugated to horse radish peroxidase (GE Healthcare) diluted at 1:100,000.

### Inoculation of ME7 or normal mouse brain homogenate into mice

All animal protocols were reviewed and approved by the Rocky Mountain Laboratories Animal Care and Use Committee (Protocols 2011–100 and 2018–046). This study was carried out in strict accordance with the recommendations in the *Guide for the Care and Use of Laboratory Animals* of the National Institutes of Health. Mice were monitored weekly and housed in HEPA-filtered cages with water and food pellets, *ad libidum*. Euthanasia was by CO2 inhalation.

Tg(WT-E1^+/+^) mice, originally made in and obtained from the laboratory of Dr. David Harris [19], are homozygous for a transgene that leads to overexpression of mouse PrP^C^ containing a unique epitope to the anti-PrP mouse monoclonal antibody 3F4 (PrP-3F4) [20]. This epitope is not present in PrP^C^ expressed in wild-type mice (S1B Fig). Tg(WT-E1^+/+^) mice, abbreviated as Tg3F4 throughout this study, were bred to *Prnp* gene knockout mice on a 129 X C57BL/6J background [21] and do not express wild-type PrP^C^ [19]. C57BL10/SnJ-Prnp^−/−^ mice knocked out for the prion protein gene *Prnp* (PrP^KO^) were used as a specificity control for PrP staining and have been described previously [10]. Tg3F4 mice (8–10 weeks old) were infected by injecting 50 μL of inoculum via the intracerebral route using a 27 gauge ½ inch needle, a process that took 3–5 seconds to complete. The same operator inoculated all animals. The ME7 inoculum was a 10% (w/v) stock consisting of brain material from wild-type RML mice clinically ill with ME7 scrapie suspended in 0.32M sucrose (stock titer = 2 x 10^7.9^ ID_50_/g). RML mice are Swiss-Webster mice that have been bred and maintained in-house at the Rocky Mountain Laboratories for decades. RML mice inoculated with ME7 develop disease with an average incubation time of 177 ± 7 days (S1A Fig) and accumulate PrP^Sc^ in the brain (S1B Fig, lanes marked ME7-3F4).

Brain homogenate was diluted to 1% in PBBS:2% fetal bovine serum (FBS), vortexed, sonicated for 2 minutes, then centrifuged for 1 minute at 3000 rpm prior to injection. The inoculum for the control group was prepared and injected exactly the same way except that a 10% (w/v) normal brain homogenate (NBH) from uninfected RML mouse brain in 0.32M sucrose was used as the starting material. At 6 hours (hrs), 24 hrs, 72 hrs, 1 week and 2 weeks post-inoculation brains were removed and prepared for immunohistochemical or immunoblot analysis as described below. For each timepoint, a total of 4 animals were inoculated and brain were removed and processed for immunohistochemistry.

For mice in which only a needle was inserted into the brain (n = 3), stereotactic inoculation was used. Briefly, mice were anesthetized with isoflurane and positioned on a stereotactic frame (David-Kopf Instruments, Tujunga, CA). Surgery and needle insertion were performed using aseptic technique. From the bregma point of reference, a 30-gauge needle (Becton Dickinson) was inserted slowly through a pre-drilled hole at a position 0.2 mm anteroposterior, 0.5 mm lateral, and 4.5 mm ventral to the skull surface. After reaching a depth of 4.5 mm the needle was slowly retracted. These coordinates were selected to avoid the ventricles. Mice received a post-operative 1.5 mg/kg injection of buprenorphine for analgesia.

### Immunohistochemistry

After transcardial perfusion with phosphate buffered saline (PBS, pH 7.2) followed by 10% normal buffered formalin (NBF), brains (n = 4 for each timepoint) were post fixed in 10% NBF. Prior to processing, the brain was bisected with a midline sagittal cut and both hemispheres submitted in one tissue cassette. The tissue was processed and embedded in paraffin with the hemispheres embedded parallel along the inferior edge. Sections (5 microns) were cut using a standard Leica microtome, placed on positively charged glass slides, and air-dried overnight at room temperature. Prior to deparaffinization, slides were heated in an oven at 60^o^ C for 20 minutes (min). The brain tissue was deparaffinized in Pro-Par clearant (Anatech Innovator), followed by rehydration in decreasing concentrations of ethanol to distilled water. For antigen retrieval, slides were immersed in 10 mM citrate buffer (pH 6.0), placed in a Biocare decloaking chamber, and treated at 120°C and 20 lb/in^2^ for 20 min. After cooling, the slides were stained using a DABMap kit and hematoxylin counterstain on an automated Discovery XT staining system (Ventana Medical Systems). The primary antibody was biotinylated 3F4 diluted 1:50 in Ventana antibody dilution buffer (Ventana Medical Systems) with an incubation time of 60 minutes.

Following antigen retrieval, some slides were stained for fluorescent microscopy as follows. All incubations were done in a closed, humidified chamber. Slides were blocked in blocking solution (2% donkey serum (DS), 1% bovine serum albumin (BSA), 0.1% fish skin gelatin (FSG), 0.1% Triton X-100, and 0.05% Tween-20 in 1X phosphate buffered saline (PBS) pH 7.2) for 30 minutes at room temperature. Excess blocking solution was removed, the first primary antibody diluted in 1% DS/PBS pH 7.2, and the slides incubated at 4°C overnight. Slides were rinsed twice for 10 min in wash solution (0.5% FSG/PBS pH 7.2) and incubated for at least 1 hr at room temperature with an Alexa Fluor 488 labeled goat anti-rabbit secondary antibody diluted 1:500 in 1% DS/PBS pH 7.2. After rinsing twice for 10 min in wash solution, slides were again incubated in blocking solution for 30 min and then incubated with biotin-conjugated 3F4 diluted 1:50 in 1% DS/PBS pH 7.2 for at least 1 hr. Following two washes for 10 min each in wash solution, slides were incubated for at least 1 hr with streptavidin-conjugated Alexa Fluor 594 diluted 1:500 in 1% DS/PBS pH 7.2. Following two 10 min rinses in wash solution, slides were coverslipped using ProLong Gold Antifade reagent with DAPI (Molecular Probes Life Technologies).

Imaging of slides was performed using widefield or confocal fluorescence microscopy. Widefield images were captured using a Nikon Eclipse 55 microscope outfitted with a Nikon Intensilight C-HGF1 fluorescence source and Digital Sight camera or with a Nikon TiE microscope outfitted with a Lumencor Spectra X illuminator and a Photometrics CoolSnapHQ2 camera. Both microscope systems were controlled using Nikon NIS-Elements Imaging Software (Nikon Instruments, Melville, NY). Experimental and control images were always acquired and analyzed with identical settings. Confocal images were acquired on a Nikon LiveScan confocal microscope using either a 60x or 100x (1.49 NA) objective, a Nikon LU-NV laser launch and a DU-897 X3 iXon camera (Andor Technology). Experimental and control images were always acquired and analyzed with identical settings. Images were analyzed using Nikon NIS-Elements and Imaris x64 software (Oxford instruments). Images were sharpened using the Gauss-Laplace sharpen tool in NIS-Elements and the figures assembled in Adobe Illustrator (Adobe Systems Mountain View, CA).

### Silver staining of axons

Axons were stained using formalin fixed, paraffin embedded tissue. The Hito Bielschowsky OptimStain Kit (Hitobiotec Corp.) was used according to the manufacturer’s instructions.

### PrP^Sc^ preparation

Tg3F4 mice were inoculated as described above (n = 4 for each timepoint), and whole brains were homogenized in PBS to 20% (w/v) with 2 x 30 sec bursts using a Minibeadbeater (Biospec Products). Tris-HCl (pH 8.5), sodium deoxycholate (DOC), and Triton X-100 were added to the homogenate to give final concentrations of 0.1 M, 1% and 1%, respectively. In one experiment, the corpus callosum and adjacent cortical tissue as well as the tissue surrounding the needle track was analyzed for protease-resistant PrP. In those animals (n = 4 for each timepoint), the needle track was visible to the naked eye at all three timepoints analyzed (6, 24 and 72 hrs). Approximately 1–2 mm of tissue was excised using a surgical scalpel and homogenized as described above.

For samples that were not proteinase K (PK) treated, 20.2 μL of sample buffer (1 M Tris-HCl pH 6.8, 10% glycerol, 6 mM EDTA, 20% sodium dodecyl sulfate (SDS)) was added to the mixture containing brain homogenate, Triton X-100, DOC and Tris-HCl pH 8.5 to a final volume of 35 μL. For detection of protease resistant PrP, PK was added to a final concentration of 63.3 μg/mL and the brain homogenate mixture was incubated for 30 min at 37°C in a circulating water bath. The PK was inactivated by the addition of 0.1 M phenylmethylsulfonyl fluoride (PMSF, final concentration 0.01 M) and 17.2 uL of 2X sample loading buffer was added for a final volume of 35 μL. All samples were boiled for 3 min. After PK treatment and boiling in sample buffer, some samples were deglycosylated using PNGase F (New England Biolabs) according to the manufacturer’s instructions.

For isolation of aggregated, protease resistant PrP by sodium phosphotungstic acid (PTA) precipitation, Tg3F4 mice were inoculated as described above (n = 4 for each timepoint) and 250 μL of 4% sarkosyl in PBS was added to 250 μL of a 10% (w/v in PBS) Tg3F4 mouse brain homogenate. The sample was mixed by pipetting and vortexing and a further 500 μL of 2% sarkosyl in PBS was added. The sample was incubated at 37°C for 30 minutes followed by the addition of 5 μL of 10 units/μL benzonase (Sigma) and 5 μL 0.2M magnesium chloride. The sample was briefly vortexed and incubated for 1 hr at 37°C followed by centrifugation at 3,000 x g for 3 min to remove cell debris. The supernatant was removed and PTA (pH 7.4) added to a final concentration of 1 mM and further incubated for 2 hrs at 37°C. The sample was then centrifuged at 16,000 x g for 30 minutes at 37°C, the supernatant removed, and 40 μL of 0.1% sarkosyl in PBS added to the pellet which was sonicated for 5 minutes. PK was added to a final concentration of 50 μg/mL and the sample incubated at 37°C for 1 hr. Protease digestion was stopped by the addition of 4 μL of 100mM Pefabloc. LDS sample buffer (4X, ThermoFisher Scientific) containing 2% β-mercaptoethanol and 4M urea was added to a final concentration of 1X and the sample was vortexed and then boiled at 100°C for 10 mins. For untreated samples, 2μL of 5mg/mL brain equivalents (by weight) was loaded per lane. For PK-treated samples, 15μL of 5 mg/mL brain equivalents was loaded per lane, an amount 7.5-fold lower than that in the untreated lanes. As a positive control for PTA precipitation of aggregated PrP-3F4, a 1:5 dilution of ME7 PrP^Sc^-3F4 brain homogenate:NBH was used.

### Western blotting

Samples were loaded onto either Novex 10% Bis-Tris or 16% Tris-glycine gels (Invitrogen). After electrophoresis, samples were transferred to polyvinylidene difluoride (PVDF) membrane (Millipore) overnight at 4^o^ C in Towbin’s buffer (0.25 M Tris, 0.192 M Glycine, 0.005% SDS, 20% methanol). Membranes were blocked with 5% milk in TBST (137 mM NaCl, 2.7 mM KCl, 19 mM Tris base, 0.1% Tween) for 1.5 hrs, then 3F4-biotin conjugated antibody diluted 1:10,000 dilution in TBST was added to the membrane for 1.5 hrs at room temperature on a shaking platform. Following a 30 min period of 3 to 4 washes in TBST, the membrane was incubated for 1.5 hrs in a 1:250,000 dilution of streptavidin-linked horseradish peroxidase in TBST at room temperature. The wash step above was repeated and membranes rinsed in distilled water before being developed with SuperSignal West Femto Maximum Sensitivity Substrate (Thermo Scientific) for detection on X-ray film.

### Protein quantitation

For quantitation of total aggregated PrP in the brains of inoculated mice, a dilution series (1 ng, 0.5 ng and 0.25 ng) of bacterially derived recombinant hamster PrP [22] was used to ensure that all blots were developed equivalently using a 20 second film exposure. Exposed film was passed through a developer (M35 XOMAT Professional; Kodiak) then digitized into 600 DPI tif images with the Windows Fax and Scan App and a flatbed scanner (LiDE 210; Cannon). The gel images were cropped and rotated in Photoshop (Adobe) before the lanes of gel images were quantified in the program UN-SCAN-IT Gel (version 7.1, Silk Scientific Corporation). Using a segmented pixel summation analysis, gel images were segmented by lane, the sum of all pixels in the PrP bands was determined, and then the pixel sums were background corrected by subtracting the product of the average pixel intensity outside each lane and the number of pixels in a lane. Corrected pixel sums for PrP in arbitrary units (a.u.) were then imported into GraphPad Prism 8 for Windows (GraphPad Software, Inc.) for plot creation.

To determine total protease-resistant PrP for each time point assayed, PK-treated and untreated samples were run on the same gel. Image acquisition and quantification was performed as described above. Total protease-resistant PrP was determined by dividing the number of pixels in a.u. in PK-treated PTA-precipitated PrP samples by the number of pixels in untreated PTA-precipitated PrP samples divided by 7.5. The number of total pixels in the untreated samples was divided by 7.5 to account for the higher mg brain equivalents loaded for those samples. The final number was then multiplied by 100 to determine the percentage of protease-resistant PrP-3F4 in PTA-precipitated samples.

### Statistics

Statistical analysis was done using a 1-way Anova with Dunnett’s multiple comparisons test in GraphPad Prism.

## Results

### Acute tissue loss following IC inoculation of brain homogenate

**Fig 1:**
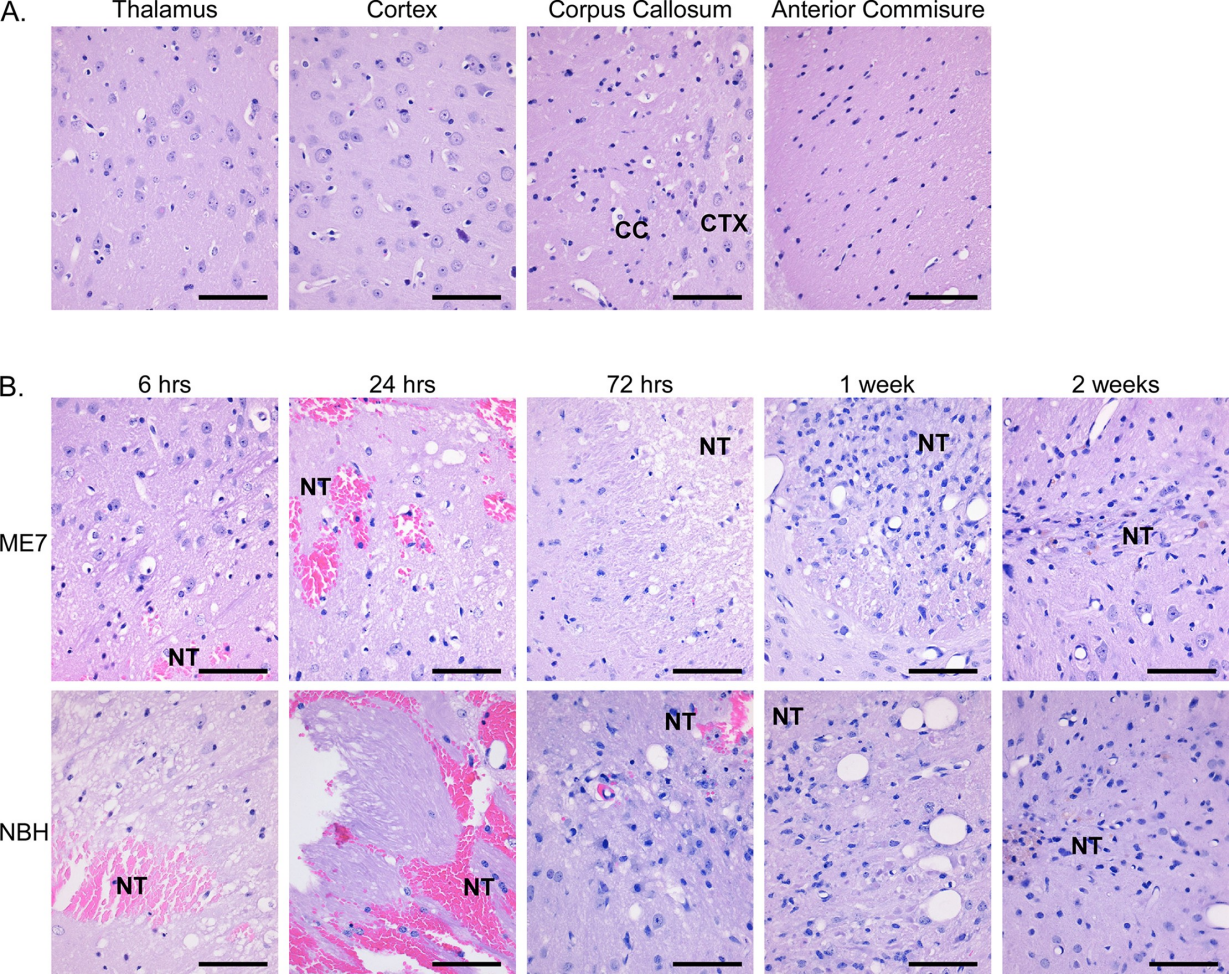
H&E stain of brain tissue of Tg3F4 mice following inoculation with ME7 prions or NBH. (A) H&E staining of different brain regions of uninoculated Tg3F4 mice. (B) H&E staining of brain tissue at different timepoints following the inoculation of mouse brain homogenate from mice infected with ME7 prions (upper row) or NBH (lower row). All sections are from the thalamus except for the ME7 6 hr timepoint (cortex/corpus callosum), the NBH 24 hour (fornix) timepoint, and the ME7 2 week timepoint (hypothalamus). Tissue loss is evident in the region surrounding the needle track. The sections shown are representative of the 4 animals analyzed at each timepoint. CC = corpus callosum; CTX = cortex; NT = needle track. Scale bar = 50 μm.

In order to analyze the response of endogenous host PrP^C^ to prion infection, Tg3F4 mice (i.e. Tg(WT-E1^+/+^) (from reference [19]) expressing 3F4 epitope tagged mouse PrP^C^ (PrP-3F4) were inoculated intracranially (IC) with normal brain homogenate (NBH) from uninfected RML mice or with brain homogenate from RML mice infected with the ME7 strain of mouse scrapie. RML mice express wild-type PrP^C^ which does not react with the 3F4 antibody (S1B Fig). At various time points from 6 hrs to 2 weeks post-infection, brains were harvested, formalin-fixed and stained either with hematoxylin and eosin (H&E) for pathological analysis or for host-specific PrP-3F4 expression using the mouse monoclonal antibody 3F4. At six hours post-inoculation with either ME7 or NBH, analysis of H&E stained brain tissue showed clear physical damage from the needle primarily in the thalamus with red blood cells and a cellular infiltrate often clearly visible when compared to uninoculated controls (Fig 1). In some sections, a gross loss of brain tissue, likely due to the volume of homogenate inoculated, was also seen and was most prominent at the 6, 24, and 72 hr timepoints (Fig 1B). Within the first 72 hours, tissue loss was evident in a zone extending several microns beyond the needle track. However, by 2 weeks post-infection, there was a reduction in damage proximal to the needle track suggesting repair of the tissue (Fig 1B). A similar pathological progression of tissue damage and repair around the needle track has also been described in hamster and sheep models of IC prion infection [23, 24]. However, we observed no significant differences between the NBH and ME7 inoculated mice. Furthermore, similar damage was observed in Tg3F4 mice where a needle was briefly inserted into the brain but no homogenate inoculated (S2A Fig). Thus, the changes observed were not related to acute prion infection but rather to a normal response of the brain to physical and/or fluid pressure damage from the IC inoculation.

### PrP-3F4 distribution and expression is altered following intracranial inoculation of brain homogenate

**Fig 2:**
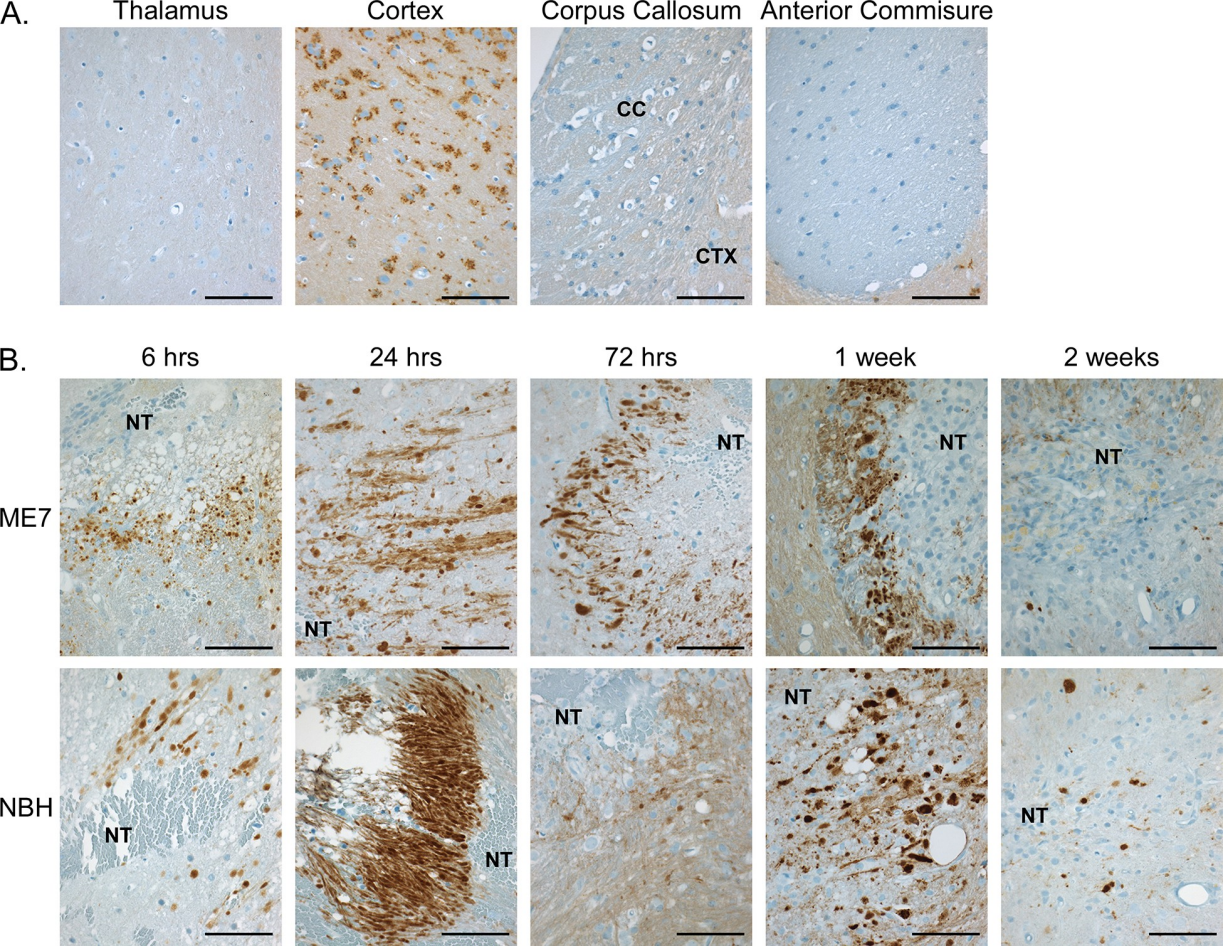
Endogenous PrP-3F4 staining in Tg3F4 mice following inoculation with ME7 prions or NBH. (A) PrP-3F4 staining in different brain regions of uninoculated Tg3F4 mice. (B) PrP-3F4 staining at different timepoints following the inoculation of mouse brain homogenate from mice infected with the ME7 strain of mouse prions (upper row) or inoculated with normal mouse brain homogenate (NBH, lower row). All sections are from the thalamus except for the ME7 6 hr timepoint (cortex/corpus callosum), the NBH 24 hr timepoint (fornix), and the ME7 2 week timepoint (hypothalamus). Sections were stained using the anti-PrP mouse monoclonal antibody 3F4 conjugated to biotin as detailed in the Materials and Methods. The sections shown are representative of the 4 animals analyzed at each timepoint. CC = corpus callosum; CTX = cortex; NT = needle track. Scale bar = 50 μm.

Next, we asked whether host PrP expression was altered in the area of damage associated with IC inoculation. In uninoculated mice, PrP-3F4 was most commonly found in a characteristic punctate, perinuclear pattern in neurons of the cortex (Fig 2A) with some synaptic stain apparent. PrP-3F4 staining was not prominent in the thalamus, corpus callosum or anterior commissure (Fig 2A). Starting at 6 hrs post-inoculation with ME7 scrapie, a different pattern of staining was observed with dense, rounded deposits as well as long streaks of PrP-3F4 positivity detected at or near the needle track (Fig 2B, top row). The specificity of the PrP-3F4 staining was confirmed using PrP^KO^ mice which showed similar tissue loss but no 3F4 positivity following inoculation with wild-type ME7 (S3 Fig). The pattern of staining we observed in the Tg3F4 mice was consistent with PrP staining that has been previously associated with dystrophic axonal processes [25] and areas of ischemic brain damage [26]. Interestingly, PrP-3F4 staining was primarily found at the margins, and not within, the areas of tissue loss associated with the needle track and dense, rounded PrP-3F4 stain was still visible near the repaired needle track 2 weeks after inoculation (Fig 2B). Although the intensity of the 3F4 stain varied from mouse to mouse, similar results were seen in Tg3F4 mice inoculated with NBH (Fig 2B, bottom row). A similar pattern of PrP-3F4 staining was also observed in Tg3F4 mice where a needle was inserted into the brain but no homogenate inoculated (S2B Fig). Overall, our data suggest that the changes in 3F4 staining were predominantly due to changes in the distribution of host PrP-3F4 in response to the inoculated brain homogenate and not to the formation of new, host-derived PrP^Sc^-3F4.

### Dystrophic axons are present in damaged regions of the brain with altered PrP-3F4 staining

Dystrophic axons are enlarged, eosinophilic axons found in multiple neurological diseases as well as aged brain that are considered to be markers of neurodegeneration [27, 28]. Consistent with the presence of dystrophic axons, areas of eosinophilic stain indicative of enlarged axonal processes were often apparent at the leading edge of the damage near the needle track (Fig 1B, S4A and S4D Fig). These same areas also stained positive for PrP-3F4 in a pattern consistent with dystrophic axons (Fig 2B, S4B and S4E Fig). Bielschowsky’s silver stain for axons confirmed the presence of swollen axonal processes and spheroids typical of dystrophic axons [28] in the same region where PrP-3F4 staining was observed (S4C and S4F Fig, arrows). At one week post-infection, dystrophic axons were still present around the needle tract (S4F Fig, arrows) and there was a clear reduction in intact axonal processes when compared to uninoculated control tissue (compare S4F Fig to S4G Fig). These data suggested that at least some of the tissue loss observed following inoculation was due to degeneration of axons in areas of damage where PrP-3F4 staining was prominent.

**Fig 3:**
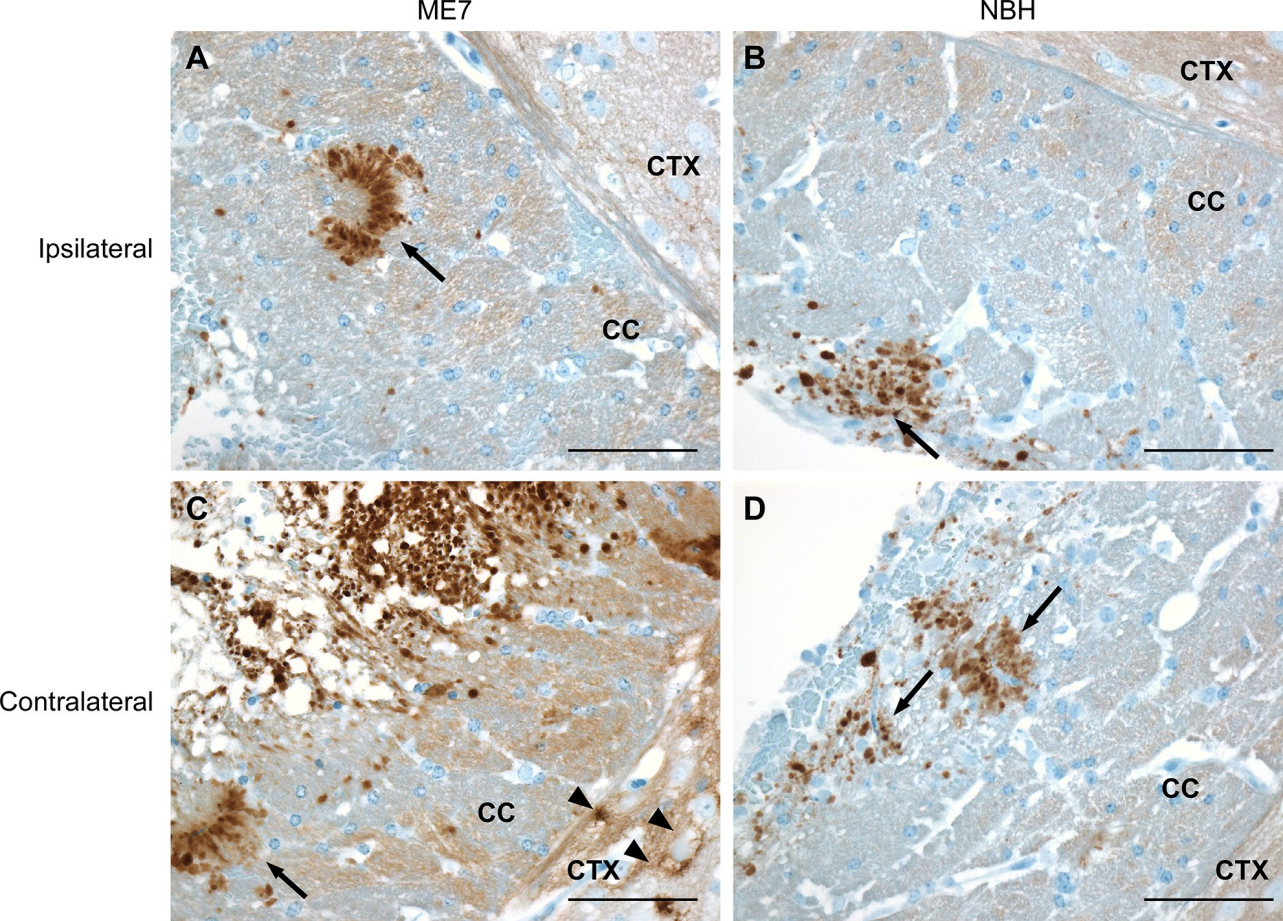
Endogenous PrP-3F4 staining in the corpus callosum and adjacent cortex in Tg3F4 mice following inoculation with ME7 prions or NBH. (A and B) PrP-3F4 staining in the corpus callosum and adjacent cortical tissue on the inoculated side of the brain (Ipsilateral) and (C and D) uninoculated side of the brain (Contralateral) 24 hrs post-inoculation. Arrows indicate PrP-3F4 staining around blood vessels. Arrowheads indicate diffuse PrP-3F4 staining around cells in the cortex distinct from that seen in normal uninoculated Tg3F4 brain (compare to Fig 2A). NBH = normal mouse brain homogenate; CC = corpus callosum; CTX = cortex. Scale bar = 50 μm.

In both ME7 and NBH inoculated mice, PrP-3F4 staining was also observed in the corpus callosum and the region of the cortex adjacent to the corpus callosum in both the ipsilateral and contralateral sides of the brain (Fig 3). Dense, rounded PrP-3F4 staining consistent with the presence of dystrophic axons was present within the corpus callosum and was often associated with areas of tissue loss (Fig 3C). In addition, intense PrP-3F4 staining was observed around blood vessels (Fig 3A–3D, arrows) in the corpus callosum and in a diffuse pattern around cells in the cortex adjacent to the corpus callosum (Fig 3C, arrowheads). This diffuse cortical PrP-3F4 stain was distinct from the perinuclear staining seen in cortical cells in uninoculated mice (Fig 2A). Given that the pattern of PrP-3F4 staining in the corpus callosum and cortex was not always associated with the needle track and was found on both sides of the brain, it is likely that at least some of the damage observed as well as the altered PrP-3F4 staining was associated with fluid pressure damage from the volume of inoculum injected.

### Changes in PrP-3F4 expression in IC inoculated mice

**Fig 4:**
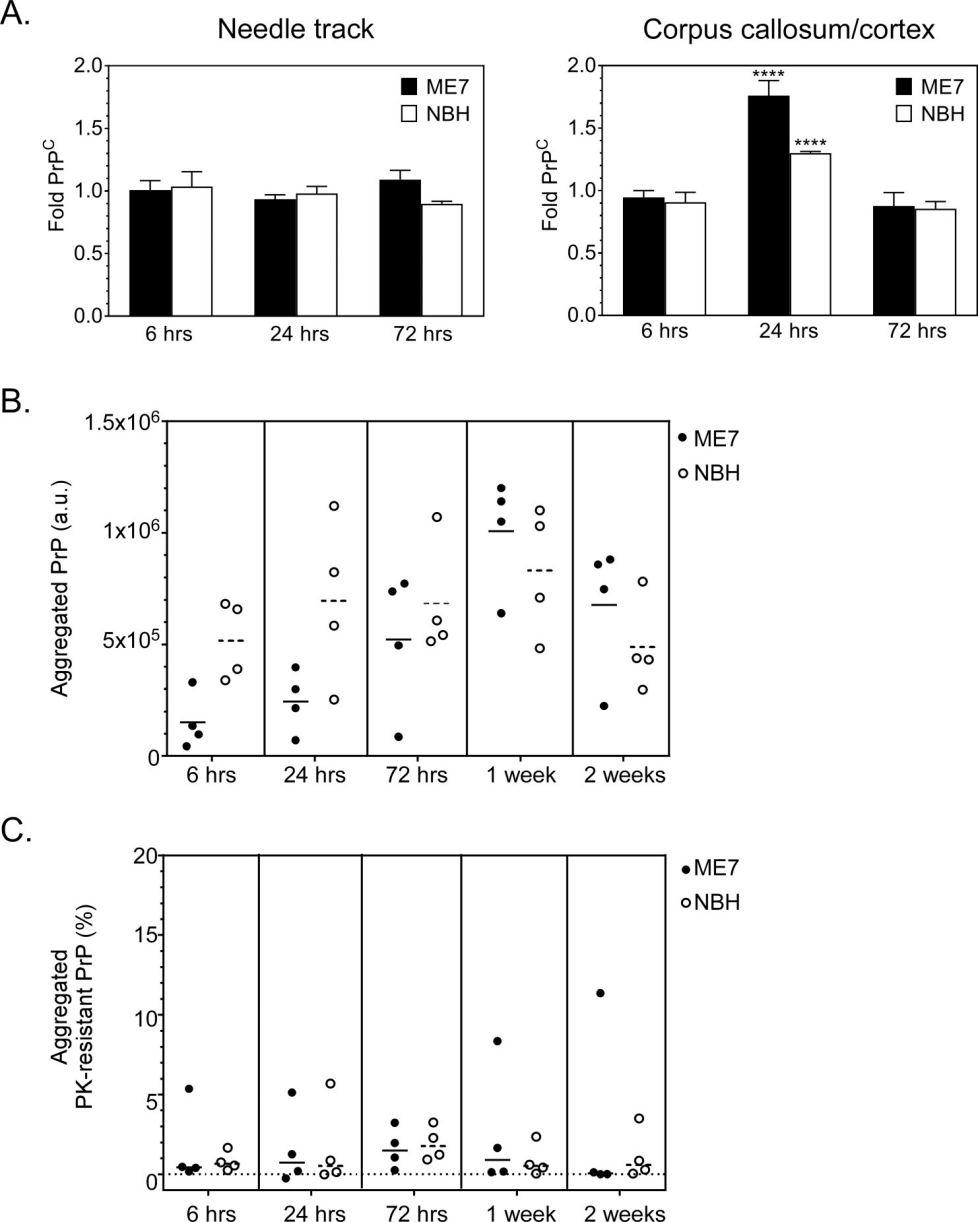
Increased expression and aggregation of PrP-3F4 in Tg3F4 mice inoculated with ME7 or NBH. (A) Fold increase in PrP-3F4 from the needle track and adjacent tissue (left panel) or corpus callosum and adjacent cortical tissue (right panel) in mice infected with ME7 prions (ME7, black bars) or normal mouse brain homogenate (NBH, white bars). The relative level of PrP-3F4 is expressed as a fold difference when compared to age-matched, uninoculated Tg3F4 mice as described in the Materials and Methods. Four mice were analyzed for each timepoint. Significant differences between control uninoculated mice and inoculated mice were calculated using the 1-way Anova multiple comparisons test. **** p = 0.003. (B) Quantitation of total aggregated PrP-3F4 PTA-precipitated from the brains of Tg3F4 mice inoculated with ME7 prions (closed circles) or NBH (open symbols). (C) Quantitation of aggregated, protease-resistant PrP-3F4 PTA-precipitated from the brains of Tg3F4 mice inoculated with ME7 prions (closed circles) or NBH (open circles). The percentage of protease-resistant PrP-3F4 was calculated as described in the Materials and methods. Examples of gels used to calculate protease-resistant PrP-3F4 for 3 different timepoints are shown in S5 Fig. For panels B and C, the short horizontal lines indicate the mean of n = 4 ME7 samples (solid line) or n = 4 NBH samples (dashed lines). The long horizontal dotted line in Panel C indicates 0 on the y-axis. a.u. = arbitrary units.

The increase in PrP-3F4 staining in areas of damage associated with the inoculation of either ME7 prions or NBH suggested an increase in overall PrP-3F4 expression. To test this hypothesis, Tg3F4 mice were inoculated IC with ME7 or NBH and at 6, 24 and 72 hrs post-inoculation the tissue surrounding the site of needle track damage in the thalamus as well as the corpus callosum and adjacent cortical tissue was dissected out. PrP-3F4 levels were determined by immunoblot and quantified by comparison to PrP-3F4 expression levels in the same brain regions from uninoculated Tg3F4 mice. There was no significant increase in the overall amount of PrP-3F4 in the tissue surrounding the needle track at any time point when compared to uninoculated Tg3F4 mice (Fig 4A, left panel). By contrast, at 24hrs there was a slight but significant increase in PrP-3F4 expression in the tissue taken from the corpus callosum/cortex (Fig 4A, right panel). By 72hrs, the expression level of PrP-3F4 in the corpus callosum and adjacent cortical region was the same as that of uninoculated Tg3F4 mice (Fig 4A, right panel). These data indicate that there was only a localized, transient increase in PrP-3F4 expression and suggest that the observed increase in PrP-3F4 staining around the needle track was not due to an increase in PrP-3F4 expression but rather to an accumulation of PrP-3F4 in the areas of damage.

### Formation of aggregated and protease resistant PrP-3F4 following IC inoculation

Acute formation of PrP^Sc^ occurs almost immediately following exposure of cells *in vitro* to prion infectivity [29–31] and is detectable *in vivo* as early as 3 days after stereotactic inoculation of prions [10]. Brain homogenate from the ipsilateral hemisphere of mice inoculated with ME7 prions or NBH was PTA-precipitated and tested by immunoblot for the presence of aggregated PrP-3F4. In ME7 inoculated mice, varying levels of 3F4-positive, aggregated, PrP-3F4 were detected at all timepoints tested (Fig 4B, S5 Fig), suggesting possible *de novo* PrP^Sc^-3F4 formation. However, aggregated PrP-3F4 was also detected at similar levels in mice inoculated with NBH (Fig 4B, S5 Fig) indicating that aggregation of PrP-3F4 was not prion-specific.

The presence of PrP^Sc^-like bands in the NBH inoculated mice suggested that the aggregated PrP-3F4 detected might not be PrP^Sc^-3F4. To look for the presence of protease-resistant PrP, PTA-precipitated PrP was digested with PK and the amount of protease-resistant PrP-3F4 was determined. As shown in Fig 4C and S5 Fig, some mice at every timepoint were positive for protease-resistant PrP-3F4 in the brain whether inoculated with ME7 or NBH. In all samples, protease-resistant PrP-3F4 appeared to migrate at or slightly above the molecular mass of PrP^Sc^-3F4 derived from clinically positive ME7 infected Tg3F4 mice (S5 Fig). Regardless of the inoculum used, the percentage of total PrP-3F4 that was protease-resistant was very low (<6%) for most samples. However, a single ME7 inoculated mouse at 1 week and 2 weeks post-inoculation had levels of protease-resistant PrP-3F4 higher than 6%, suggesting *de novo* formation of PrP^Sc^-3F4 (Fig 4C). Thus, following IC inoculation host PrP^C^ can aggregate and acquire protease resistance in damaged and degenerating regions of the brain but only in prion-infected mice are there signs that it can propagate.

**Fig 5:**
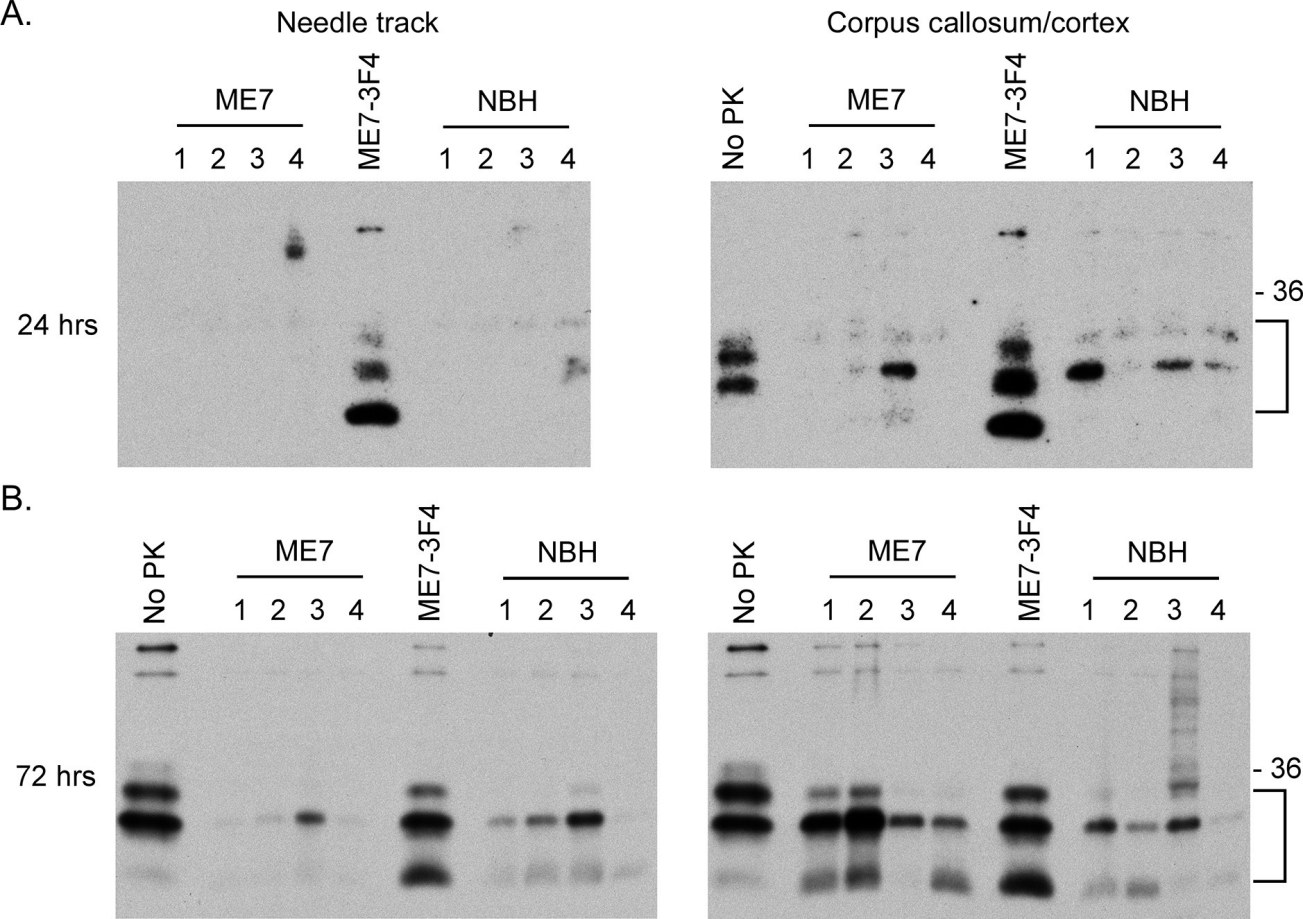
Protease resistant PrP-3F4 is present in the brains of mice inoculated with ME7 prions or NBH. Brain tissue dissected at (A) 24 hrs or (B) 72 hrs post-inoculation from either the tissue adjacent to the needle track (needle track, left panels) or the corpus callosum and adjacent cortical tissue (corpus callosum/cortex, right panels) was homogenized, PK-digested, and PNGaseF treated as described in the Materials and Methods. For each inoculum, tissue from 4 individual mice was assayed (lanes numbered 1–4). Lanes marked No PK show PrP-3F4 in brain tissue from uninoculated Tg3F4 mice that was PNGaseF treated but not digested with PK. Lanes labeled ME7-3F4 show PrP^Sc^-3F4 in PK-digested, PNGaseF treated brain tissue from a clinically positive Tg3F4 mouse inoculated with ME7 prions. The 36kDa molecular mass marker is shown on the right and the bracket indicates PrP-3F4 specific bands. Blots were developed using a biotinylated form of the anti-PrP mouse monoclonal antibody 3F4.

In order to determine if there was any PrP^Sc^-3F4 directly associated with the areas of 3F4 staining shown in Figs 2 and 3, the tissue surrounding the site of needle track damage in the thalamus as well as the corpus callosum and adjacent cortical tissue was dissected out 24 and 72 hrs after IC inoculation with either ME7 or NBH. Following digestion with proteinase K and treatment with PNGase F to remove complex glycans, the samples were analyzed by immunoblot using the 3F4 antibody. Samples of tissue surrounding the needle track were negative for protease-resistant PrP-3F4 at 24 hours post-inoculation (Fig 5A, left panel). However, consistent with the data in Fig 4B and 4C, by 72 hours post-infection most mice inoculated with either ME7 or NBH showed low levels of protease-resistant PrP-3F4 (Fig 5B, left panel) in the tissue surrounding the needle track. The most prominent band at ~21kDa was more abundant in NBH inoculated mice which also had detectable levels of an ~17kDa truncated form of PrP-3F4. Both PrP-3F4 bands migrated identically to PNGaseF treated PrP^Sc^-3F4 from clinically positive ME7-infected Tg3F4 mice (Fig 5, lanes marked ME7-3F4), suggesting that the molecular mass differences observed in S5 Fig were likely due to differences in PrP-3F4 glycosylation.

In contrast to tissue from the needle track, tissue dissected from the corpus callosum/cortex region from at least some of the mice inoculated with either ME7 or NBH was positive at 24 hours for protease-resistant PrP-3F4 (Fig 5A, right panel). These data are consistent with the data in Fig 4A demonstrating that higher levels of PrP-3F4 were present in the corpus callosum/cortex region 24 hours post-inoculation when compared to the region surrounding the needle track in the thalamus. By 72 hours, all samples were positive for protease resistant PrP-3F4 with a banding pattern very similar to that of PrP^Sc^-3F4 from ME7 infected Tg3F4 mice (Fig 5B, right panel). The greater abundance of protease-resistant PrP-3F4 in two of the 4 ME7 inoculated Tg3F4 mice is consistent with the formation of at least some *bona fide* PrP^Sc^ as early as 72hrs post-inoculation [10].

### PrP-3F4 does not colocalize with astrocytes or microglia in damaged regions of the brain following IC inoculation

**Fig 6:**
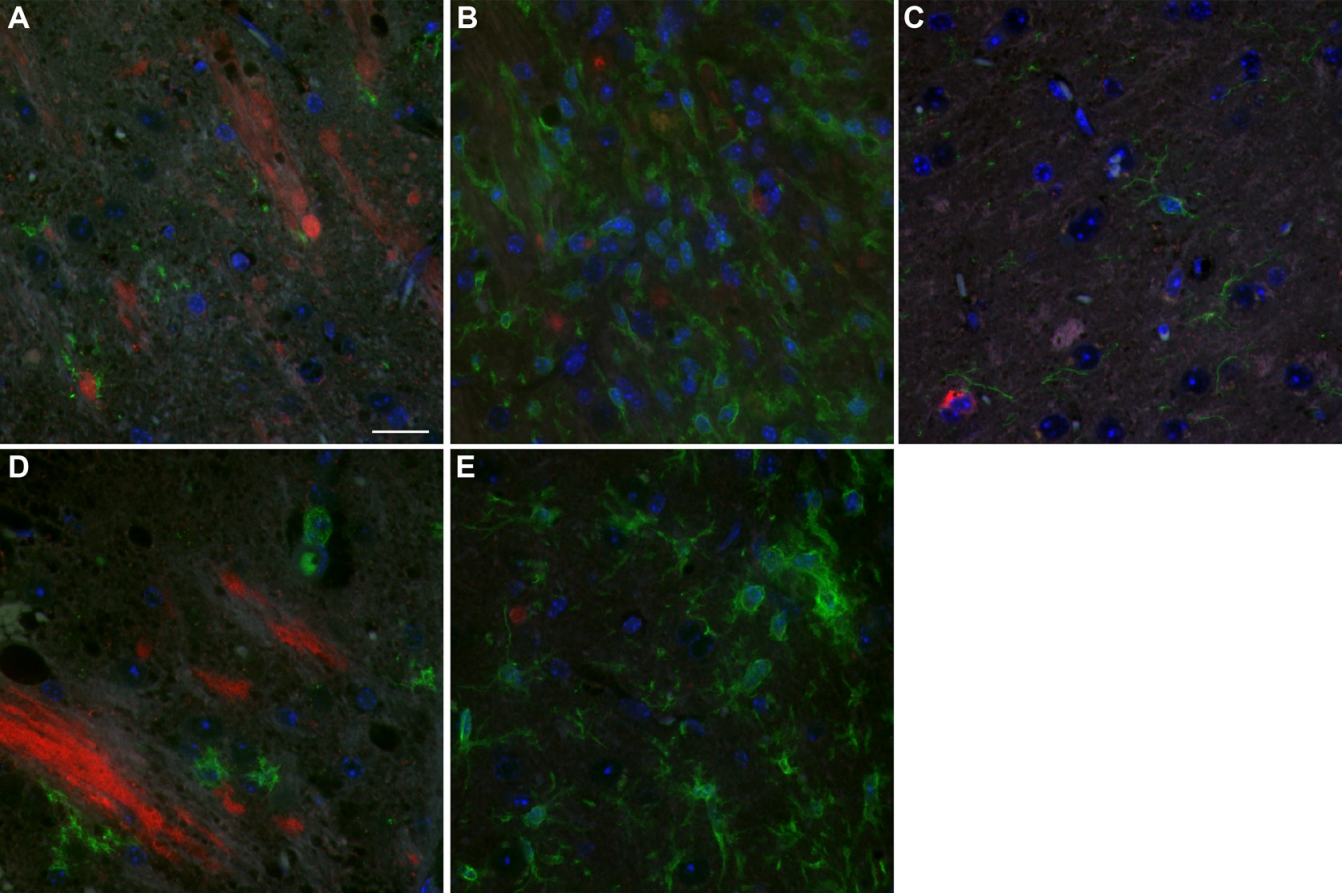
PrP-3F4 does not colocalize with microglia following IC inoculation of Tg3F4 mice with ME7 prions or NBH. PrP-3F4 (red) and Iba1(green) in the hypothalamus of NBH (A and B) or thalamus of ME7 (D and E) inoculated mice 24 hrs (A and D) and 2 weeks (B and E) post inoculation. (C) Thalamus of uninoculated Tg3F4 mouse. PrP-3F4 was detected using a biotinylated form of the anti-PrP mouse monoclonal antibody 3F4. The no primary control is shown in Fig 7C. DAPI stain for nuclei = blue; Scale bar = 20 μm.

**Fig 7:**
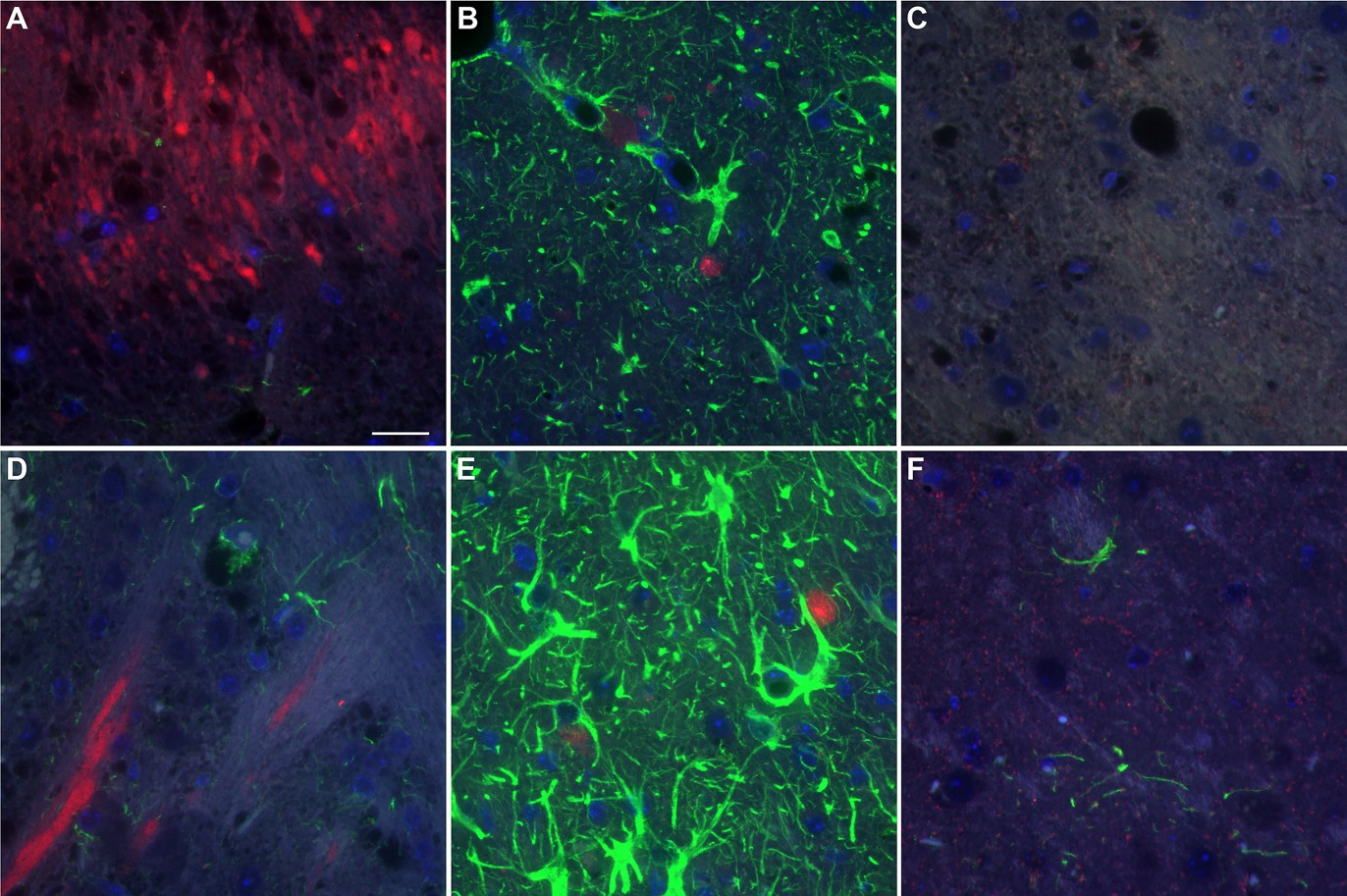
PrP-3F4 does not colocalize with astrocytes following IC inoculation of Tg3F4 mice with ME7 prions or NBH. PrP-3F4 (red) and GFAP (green) in the hypothalamus of NBH (A and B) or thalamus of ME7 (D and E) inoculated mice 24 hrs (A and D) and 2 weeks (B and E) post inoculation. (C) No primary control. (F) Thalamus of unninoculated Tg3F4 mouse. PrP-3F4 was detected using a biotinylated form of the anti-PrP mouse monoclonal antibody 3F4. DAPI stain for nuclei = blue; Scale bar = 20 μm.

It is well known that a neuroinflammatory response involving the recruitment of microglia and astrocytes is triggered following physical damage to the brain [32]. Iba1 positive microglia were detectable at the edges of the needle track 24 hrs after inoculation of either NBH (Fig 6A) or ME7 (Fig 6D) as were GFAP positive astrocytes (Fig 7A and 7D) at levels similar to that seen in uninoculated Tg3F4 mice (Fig 7F). However, by 2 weeks post-inoculation, both Iba1 and GFAP positive cells were prominent around the needle track in both NBH (Figs 6B and 7B) and ME7 (Figs 6E and 7E) inoculated mice. There was no obvious co-localization of PrP-3F4 with Iba1 or GFAP at either timepoint (Figs 6 and 7, red fluorescence), indicating that neither microglia nor astrocytes were the primary source of the protease-resistant PrP-3F4 detected by immunoblot.

### PrP is localized intra-axonally and/or with myelin near the region of damage following IC inoculation

**Fig 8:**
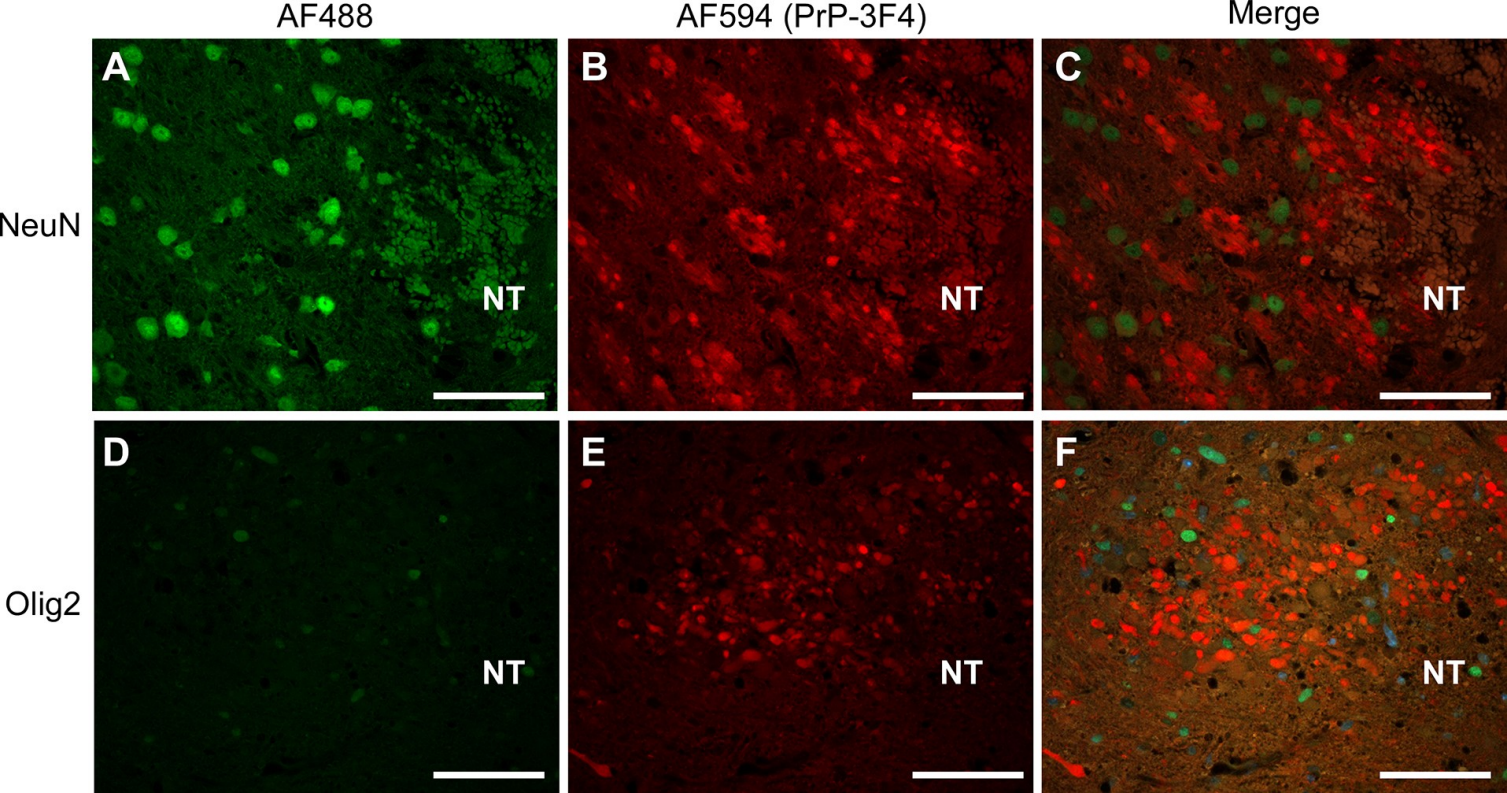
Altered PrP-3F4 staining does not co-localize with neuronal or oligodendrocyte nuclei in the brains of Tg3F4 mice inoculated with ME7 prions. (A) Area surrounding the needle track 24 hrs post-inoculation co-stained for neurons using the neuron specific antibody NeuN (AF488) and (B) PrP-3F4 (AF594). (C) Merged image of NeuN and PrP-3F4 staining. (D) Area surrounding the needle track 72 hours post-inoculation co-stained for oligodendrocytes using the antibody Olig2 (AF488) or (E) PrP-3F4 (AF594). (F) Merged image of Olig2 and PrP-3F4 staining. PrP-3F4 was detected using a biotinylated form of the anti-PrP mouse monoclonal antibody 3F4. Sections stained with NeuN are from the thalamus while sections stained with Olig2 are from the hypothalamus. AF488 = Alexa Fluor 488 goat anti-rabbit secondary antibody; AF594 = Alexa Fluor 594 secondary antibody conjugated to streptavidin; NT = needle track. Scale bar = 50 μm.

In neurons, PrP^C^ is primarily found in the Golgi apparatus and on the cell surface [33, 34] but there is some evidence that PrP^C^ can also be found in oligodendrocytes [35, 36]. In order to determine if the PrP-3F4 stain observed around the areas of damage was associated with neurons or oligodendrocytes, brain sections were co-stained with the anti-PrP antibody 3F4, an antibody to the neuron-specific protein NeuN which stains neuronal nuclei, or an antibody to Olig2, a protein expressed in the nuclei of oligodendrocytes. Since there was no difference in the pattern of PrP-3F4 staining in ME7 or NBH inoculated mice (Fig 2), only ME7 inoculated Tg3F4 mice are shown. In the area surrounding the needle track, PrP-3F4 positive stain was clearly distinct from both the NeuN and Olig2 markers (Fig 8). Therefore, the distinctive PrP-3F4 staining observed around the needle track did not appear to be associated with the cell bodies of either neurons or oligodendrocytes.

**Fig 9:**
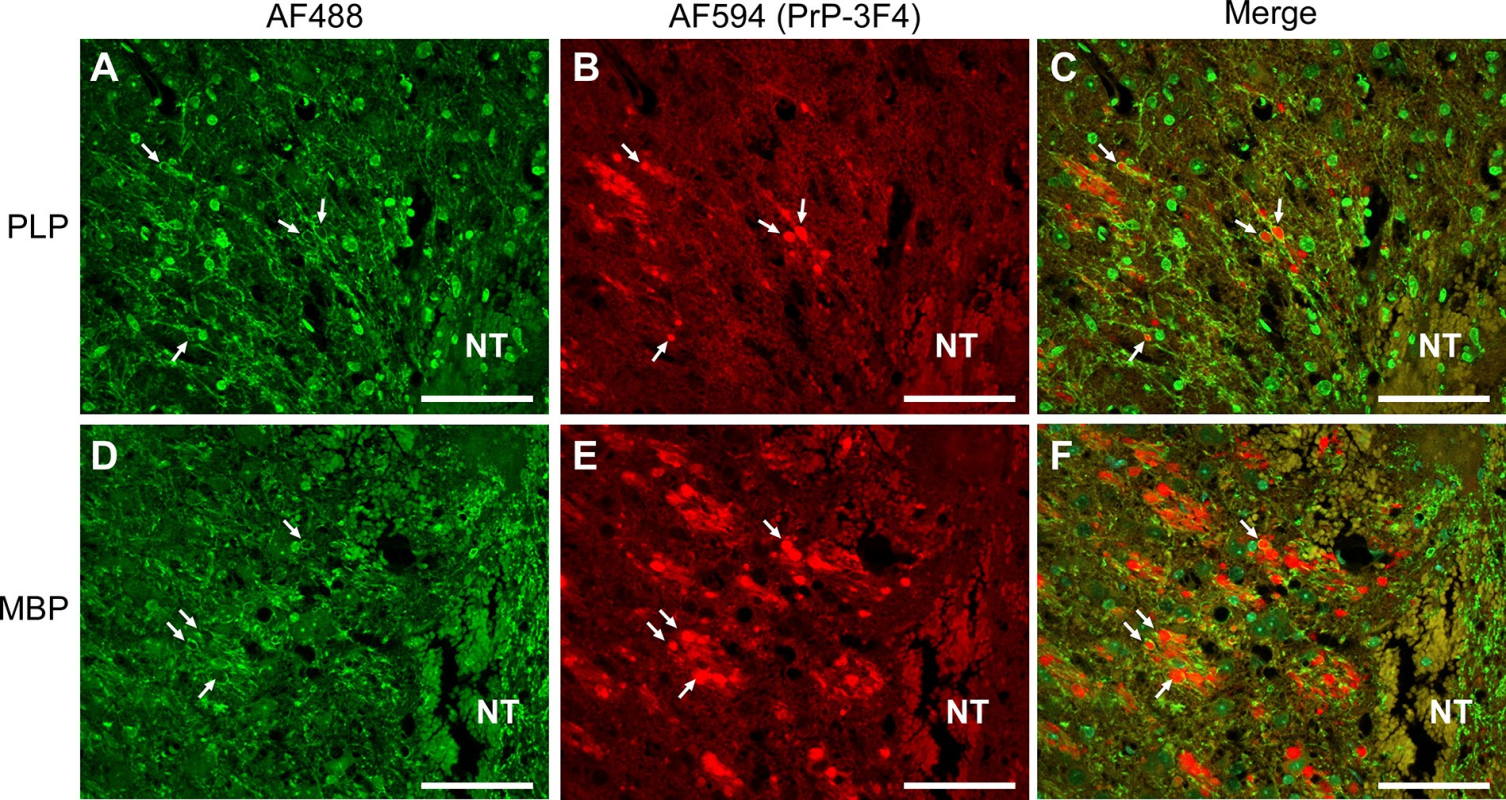
Intra-axonal localization of PrP-3F4 in the brain of Tg3F4 mice inoculated with ME7 prions. Damaged brain tissue surrounding the needle track in the thalamus of Tg3F4 mice 48 hrs post-inoculation with ME7. Sections were co-stained with antibodies to either (A) PLP and (B) PrP-3F4 or (D) MBP and (E) PrP-3F4. Panels C and F show the merged images of the preceding panels. Arrows show where PrP-3F4 staining is surrounded by either PLP or MBP staining, indicating intra-axonal localization of PrP-3F4. PrP-3F4 was detected using a biotinylated form of the anti-PrP mouse monoclonal antibody 3F4. AF488 = Alexa Fluor 488 goat anti-rabbit secondary antibody; AF594 = Alexa Fluor 594 secondary antibody conjugated to streptavidin; NT = needle track. Scale bar = 50 μm.

The PrP-3F4 staining we observed is consistent with previous work associating dense, rounded PrP^C^ staining with dystrophic axonal processes in non-prion neurodegenerative diseases [25], atypical scrapie [37], and areas of ischemic brain damage [26]. Thus, it was possible that PrP-3F4 was localizing to axons in our IC inoculated mice. We therefore co-stained brain sections from ME7-inoculated Tg3F4 mice for PrP-3F4 and the most abundant myelin-associated proteins found in the central nervous system (CNS): proteolipid protein (PLP) and myelin basic protein (MBP) [38]. As shown in Fig 9, both PLP (Fig 9A–9C) and MBP (Fig 9D–9F) was found surrounding some of the dense, round PrP-3F4 stain at the edges of the needle track in the thalamus (Fig 9, arrows). Confocal analysis of the tissue at a higher magnification confirmed that PrP-3F4 was often surrounded by PLP (S6 Fig). This pattern of staining is consistent with localization of PrP-3F4 within axons, at least some of which are myelinated, and was only observed in regions of damage and degeneration associated with the IC inoculation.

**Fig 10:**
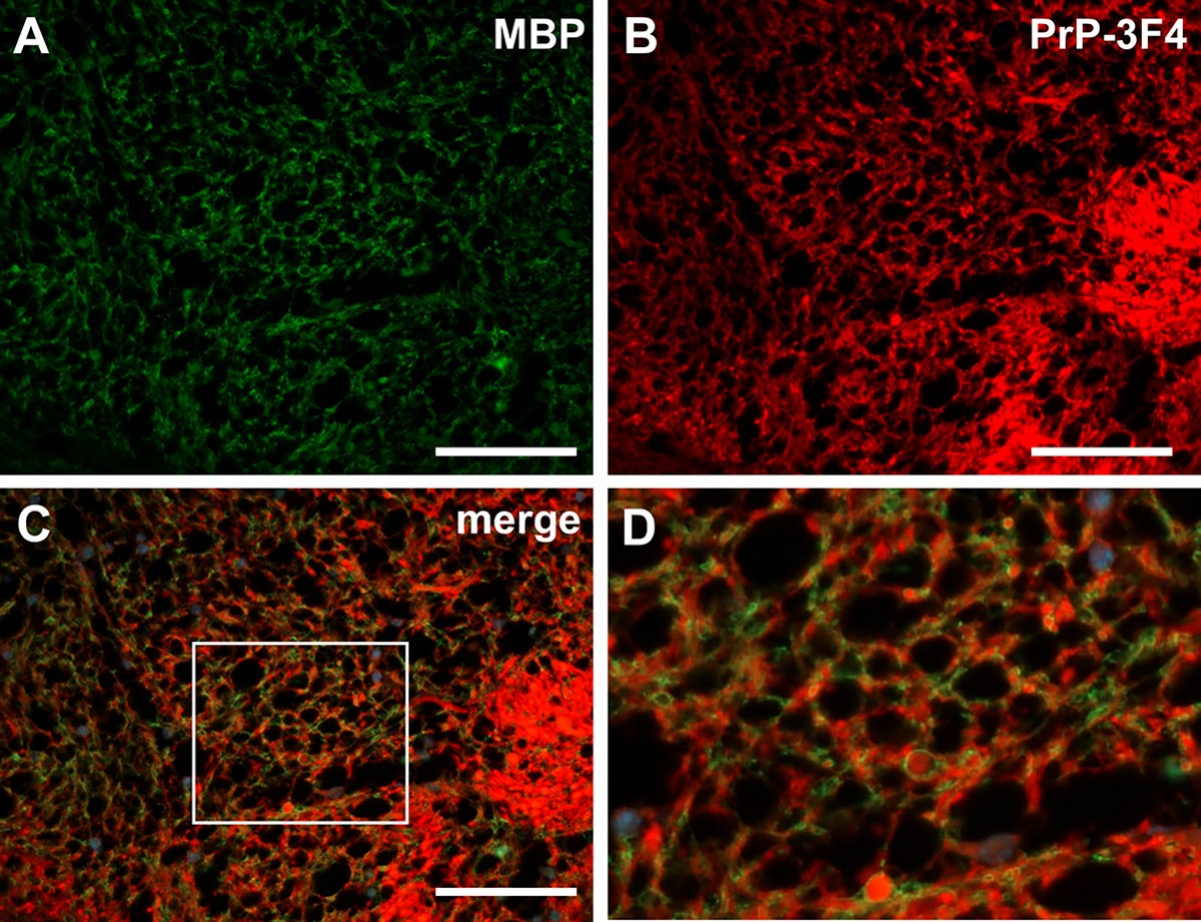
PrP-3F4 is localized either intra-axonally or within the myelin of axons in an anterior commissure damaged following inoculation of NBH. Widefield immunofluorescence microscopy of the anterior commissure 24 hrs after inoculation with NBH. Tissue was co-stained with antibodies to (A) MBP and (B) PrP-3F4. Dense, rounded staining consistent with intra-axonal PrP is visible in panel B. (C) Merged image of Panels A and B. (D) Close-up view of area enclosed by the box in (C) showing degenerated axons surrounded by both MBP and PrP-3F4 staining. Scale bar = 50 μm.

**Fig 11:**
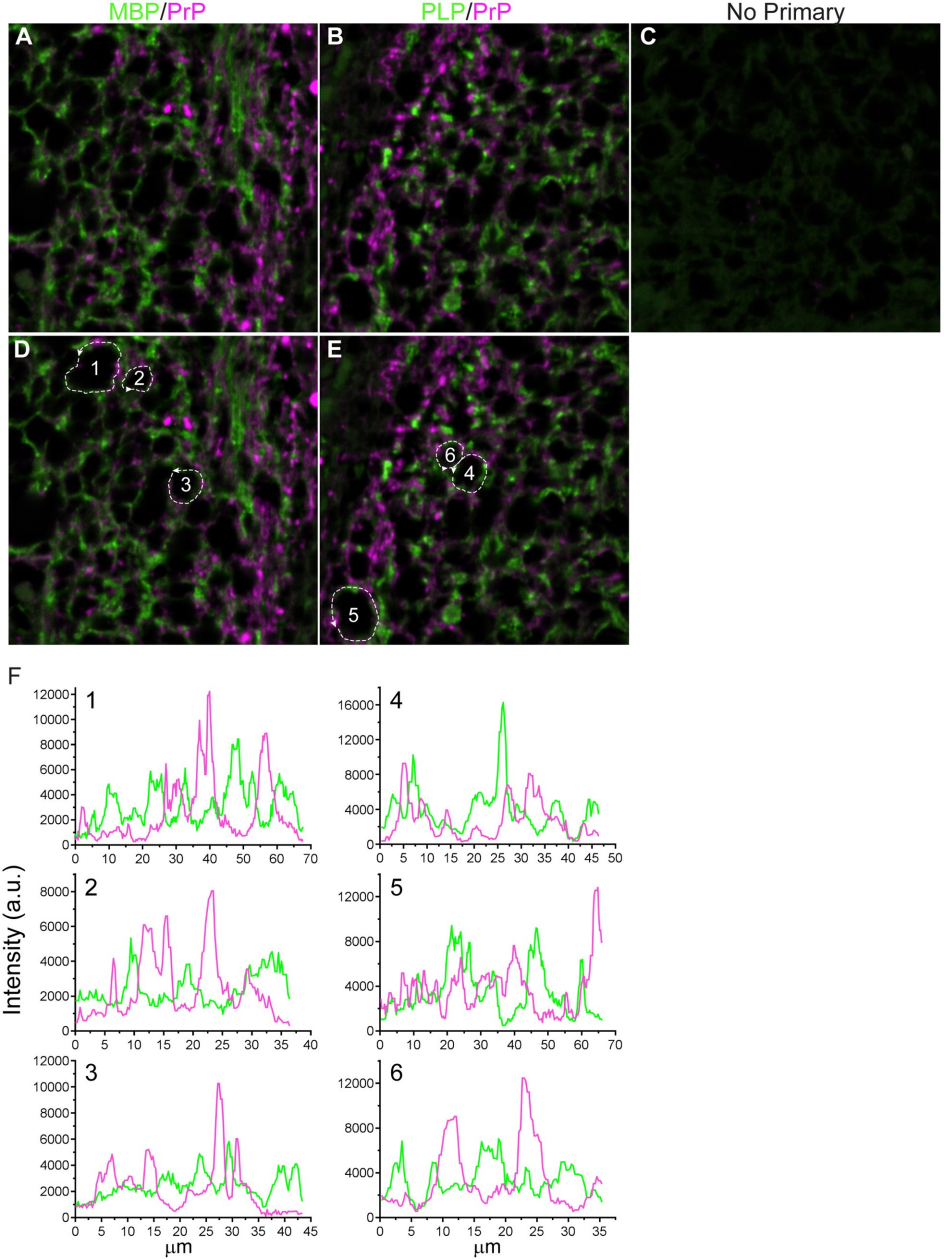
PrP-3F4 in myelin localizes to membrane domains distinct from MBP and PLP. Confocal microscopy of the anterior commissure 24 hrs after inoculation with NBH. All panels were taken using the same settings. (A) Tissue was co-stained with antibodies to MBP (green) and PrP-3F4 (magenta). (B) Tissue was co-stained with antibodies to PLP (green) and PrP-3F4 (magenta). (C) no-primary control. (D) and (E) are the same as panels A and B but with dashed lines (pixel width = 1) to designate pixels around degenerated axons where fluorescence intensity was quantified in F. (F) Histograms of signal intensity (in arbitrary units) for each pixel designated by the dashed lines in panels D and E. Numbers in each histogram correspond to the numbers in panels D and E. a.u. = arbitrary units.

PrP-3F4 positivity was often in close proximity to PLP and MBP staining in the white matter tracts of several inoculated mice, including the fornix and the anterior commissure (AC). This was seen most clearly in an AC damaged directly by the needle where there was axonal loss (Fig 10A) coincident with PrP-3F4 staining (Fig 10B and 10C). However, the pattern of PrP-3F4 staining varied. Dense, rounded PrP-3F4 stain consistent with localization within the axon was prominent in some areas of the AC (Fig 10B and 10C). In other areas, PrP-3F4 was present in close proximity to MBP and thus appeared to be associated with the myelin sheath (Fig 10C and 10D). Confocal microscopy confirmed that PrP-3F4 could be found localized to the same plane as both PLP and MBP (Fig 11A–11C). Interestingly, PrP-3F4 staining appeared to be present in domains distinct from those of both PLP and MBP, although there were some areas of minor overlap (Fig 10D–10F). Our results suggest that, as a result of axonal injury, PrP^C^ may accumulate intra-axonally as well as within the myelin sheath as the axon degenerates.

## Discussion

In this study, we used transgenic mice expressing a uniquely epitope-tagged PrP molecule (PrP-3F4) to monitor host PrP^C^ and the *de novo* formation of host-derived PrP^Sc^ following IC inoculation of prions. In some mice, we detected increased levels of protease-resistant host PrP by immunoblot as early 72 hrs post-inoculation, a result consistent with a previous study [10] and suggestive of *de novo* PrP^Sc^ formation. However, unlike the previous study [10], we also detected similar levels of aggregated and protease-resistant PrP in our NBH controls at 72 hours (Fig 4C). Thus, due to changes in both the cellular distribution and biochemical properties of host PrP^C^ as a result of the inoculation itself, we were unable to definitively distinguish host derived PrP^Sc^ from host PrP^C^ at 72 hours. At one and two weeks post-infection we did find some evidence of PrP^Sc^ propagation, but that was only in a single mouse at each timepoint. Our study highlights the difficulties in distinguishing newly formed PrP^Sc^ *in vivo*, even when using uniquely tagged PrP^C^ molecules, and suggests that the response of host PrP^C^ to intracranial inoculation may confound the biochemical identification of *bona fide* PrP^Sc^ during early prion infection.

The pattern of PrP-3F4 staining we observed surrounding the inoculation site is similar to that observed in a survey of post-mortem human brain tissue [39], in hypoxic human brain tissue from cases of cerebral ischemia [26] and stroke [40], and in brain tissue of sheep with various neurological diseases [25]. Thus, it was neither specific to prion infection nor solely an artifact of intracranial inoculation of brain homogenate. Based on the morphology and pattern of staining, previous studies concluded that PrP^C^ was most likely accumulating intra-axonally in dystrophic, swollen axons following damage or insult to the brain [25, 26, 39]. Our results are consistent with this conclusion and further demonstrate that the rounded, dense deposits of PrP-3F4 stain are sometimes surrounded by both PLP and MBP, indicating that this pattern of PrP staining can be associated with the presence of intra-axonal PrP in myelinated axons.

In previous studies of post-mortem human brain tissue [26, 39] and diseased sheep brain [25], it was unclear whether PrP^C^ associated with dystrophic axons was due to increased PrP^C^ expression [41] or PrP^C^ accumulation as a result of axonal damage. By contrast, it has been reported that PrP^C^ levels are at least transiently increased following acute stroke [40] and focal cerebral ischemia [42]. Consistent with these latter observations, we did detect a transient increase in PrP-3F4 (Fig 4A) in areas where PrP^C^ staining around blood vessels was apparent (Fig 3). However, we detected no increase in total PrP-3F4 expression in the area surrounding the needle track in either ME7 or NBH inoculated mice over the first 72 hours following inoculation (Fig 4A). This suggests that the increase in PrP-3F4 staining in these latter areas was due to a localized accumulation of PrP-3F4 and not increased expression.

PrP^C^ is known to move down the axon via fast axonal transport [43]. As previously hypothesized [39], the increased PrP staining we observed following IC inoculation could thus be a consequence of the inoculation disrupting fast axonal transport and the resultant accumulation of PrP-3F4 within the damaged axon. Disruption in axonal transport is also a known consequence of traumatic brain injury (TBI) which results in diffuse axonal injury (DAI) whereby proteins can accumulate within the damaged axon (see [44] for review). Interestingly, several proteins associated with other neurodegenerative diseases of protein misfolding accumulate in damaged axons including amyloid precursor protein (APP), which is used as a diagnostic marker of DAI [45], its cleavage product Aβ, presinilin-1, phosphorylated tau, and the Parkinson’s disease associated protein α-synuclein [46]. Our data strongly suggest that PrP^C^ is another protein associated with neurodegeneration that can accumulate within axons following damage to the brain.

It has been suggested that locally higher protein concentrations in disrupted axons can lead to the formation of potentially damaging protein aggregates, neurodegeneration, and disease [44]. For example, the release of accumulated Aβ as the axon breaks down could lead to the formation of Aβ plaques in the area of damage [44]. In support of this hypothesis, deposition of Aβ in plaques has been observed following TBI [47] and there is an increased risk of Alzheimer’s disease associated with head trauma [48, 49]. Our data clearly show that host PrP^C^ also forms aggregates following damage to the brain (Fig 4B), a small percentage of which are protease-resistant (Fig 4C).

Our results demonstrating increased PrP aggregation and protease-resistance following brain injury may suggest a potential mechanism for the origin of sCJD infection. Increased protease resistance is indicative of a change in PrP^C^ conformation. If the intra-axonal accumulation of PrP^C^ associated with stroke [40], ischemia [26, 42] or other forms of brain damage also leads host PrP^C^ to aggregate and acquire protease resistance, PrP^C^ could conceivably acquire a conformation that transforms it into infectious PrP^Sc^. Our data thus raise the intriguing but speculative possibility that brain injury may initiate events that if not resolved could, in rare instances, lead to productive prion infection and the onset of sCJD. Consistent with this hypothesis, sCJD has recently been reported in 3 cases of chronic traumatic encephalopathy (CTE), a neurodegenerative disease associated with repeated brain trauma [50].

Intra-axonal PrP^C^ staining was always found at the edge of areas of damage surrounding the needle track (Fig 2 and S2 Fig) or in areas of axonal loss (Fig 10 and S4 Fig) suggesting a potential role for PrP^C^ in the cellular and axonal loss associated with damage to the brain. At least one paper has shown that PrP^C^ expression level correlates with increasing levels of phosphorylated tau following TBI, suggesting that PrP^C^ may be involved in the pathological changes observed following brain injury [51]. Other studies using transgenic mice over-expressing PrP^C^ have shown that, in the absence of brain injury, intra-axonal accumulation of aggregated and partially protease-resistant PrP^C^ is associated with a progressive neurological disorder and synaptic dysfunction [52–54]. There are also data suggesting that aggregated axonal PrP^C^ may be directly toxic to axons [55]. Thus, it’s possible that the aggregated intra-axonal PrP we detected might contribute to the axonal and cellular loss we observed as a result of IC inoculation.

In addition to the intra-axonal accumulation of PrP-3F4 following intracranial inoculation, we also found evidence that PrP-3F4 may be present in the myelin sheath in domains that appeared to alternate with, and be largely distinct from, both PLP and MBP (Fig 11F). This pattern of PrP-3F4 staining was most obvious in white matter tracts (Figs 10 and 11) in places where the axons appeared to have degenerated and intra-axonal PrP staining was absent. Our results are consistent with data from other studies demonstrating that PrP^C^ can be found in oligodendrocytes [35, 36], the myelin sheath [56], and preparations of purified myelin [38, 57]. Functionally, PrP^C^ in both neurons and oligodendrocytes appears to be involved in myelin maintenance in the CNS [57–60] while in the peripheral nervous system myelin integrity is dependent on PrP^C^ expression in neurons [61]. This function of PrP^C^ maps to a hydrophobic region of the molecule [57–59, 61, 62] that includes at least part of a known transmembrane sequence [63] Our data are consistent with a role for PrP^C^ in myelin maintenance or remodeling following brain injury that may involve aggregation of PrP^C^ in the axon and/or insertion of PrP^C^ into the myelin membrane, either of which may be dependent upon its hydrophobic transmembrane domain.

## Supporting information

S1 Fig Incubation time, survival curve, and PrP immunoreactivity of the mouse prion strain ME7 inoculum. (A) Survival curve of RML mice (n = 11) inoculated IC with the ME7 strain of mouse scrapie. The average incubation time to disease in days ± SD is shown. The mice were inoculated using the same procedure and inoculum as that used for these studies. (B) Western blot analysis of PrP in normal brain homogenate (NBH) or ME7 infected brain homogenate (ME7) using the anti-PrP monoclonal antibodies 6D11 (left panel) or 3F4 (right panel). Samples were either undigested (-) or digested (+) with 63 μg/ml of proteinase K (PK). As a positive control for the 3F4 antibody, brain homogenate from an ME7 infected Tg3F4 mouse was used (ME7-3F4). Molecular mass markers are shown on the right.
(TIF)

S2 Fig H&E and endogenous PrP-3F4 staining in Tg3F4 mice following stereotactic insertion of a needle into the brain. (A) H&E staining of the corpus callosum 24 hrs after stereotactic needle insertion. (B) PrP-3F4 staining in the corpus callosum 24hrs after stereotactic needle insertion. The tissue was stained using the anti-PrP mouse monoclonal antibody 3F4 conjugated to biotin as detailed in the Materials and Methods. NT = needle track. Scale bar = 50 μm.
(TIF)

S3 Fig Lack of PrP-3F4 staining in PrPKO mice inoculated IC with ME7 prions. Left side panels: H&E staining of the thalamus 24 hrs and 2 weeks after inoculation with ME7 mouse prions. Right side panels: Thalamus 24 hrs and 2 weeks after inoculation with ME7 mouse prions. The tissues were stained using the anti-PrP mouse monoclonal antibody 3F4 conjugated to biotin as detailed in the Materials and Methods. Scale bar = 50 μm.
(TIF)

S4 Fig Dystrophic axons in damaged areas of the brain following IC inoculation of NBH or ME7 prions. (A) H&E stain of fornix 24 hrs after inoculation of NBH. The field of view is the same as the NBH 24 hr timepoint in Fig 1B. (B) PrP-3F4 stain of fornix 24 hrs after inoculation of NBH. The field of view is the same as the NBH 24 hr timepoint in Fig 2B. (C) Bielschowsky’s silver stain for axons in fornix 24 hrs after inoculation of NBH. (D) H&E stain of thalamus one week post-inoculation with NBH. The field of view is the same as the NBH one week timepoint in Fig 1B. (E) PrP-3F4 stain of thalamus one week post-inoculation with NBH brain homogenate. The field of view is the same as the NBH one week timepoint in Fig 2B. (F) Bielschowsky’s silver stain for axons in the thalamus one week after inoculation with NBH. (G) Bielschowsky’s silver stain for axons in the thalamus of an uninoculated Tg3F4 mouse. In Panels A, B, D and E black arrows indicate examples of swollen dystrophic axons and spheroids. In Panels C and F, white arrowheads indicate examples of swollen dystrophic axons and spheroids Scale bar = 50 μm.
(TIF)

S5 Fig PrP-3F4 aggregates and acquires protease-resistance following inoculation of ME7 prions or NBH. Western blots of PrP-3F4 PTA-precipitated from the ipsilateral side of the brain 24 hrs, 72 hrs, or 2 weeks after inoculation with ME7 prions or NBH. For each inoculum, tissue from 4 individual mice was assayed (lanes numbered 1–4). Lanes marked PK- show the total amount of PrP-3F4 PTA precipitated from the brain tissue. Lanes labeled PK+ show the amount of PK-resistant PrP-3F4 present in the PTA-precipitate. ME7-3F4 = PrP^Sc^-3F4 PTA precipitated from a 1:5 dilution of ME7-3F4 prions into NBH-3F4. NBH-3F4 = PTA precipitate from an uninoculated Tg3F4 mouse brain. The bracket indicates the PrP-3F4 specific bands used for the quantitation shown in Fig 4B and 4C. Blots were developed using a biotinylated form of the anti-PrP mouse monoclonal antibody 3F4. Molecular mass markers are shown on the right side of each panel.
(TIF)

S6 Fig PrP-3F4 is present within myelinated axons 24 hours following IC inoculation of Tg3F4 mice with ME7 prions. Two different fields of view from the thalamus are shown. Panels A and D show PrP-3F4 staining (red), panels B and E show PLP staining (green), and panels C and F show a merge of the two preceding panels. Yellow arrows indicate PrP-3F4 stain surrounded by PLP. The scale bar in panel A is 5 μm and applies to all panels.
(TIF)

S1 Table Primary antibodies used for immunohistochemistry and their specificities. (DOCX)
