# Supplementary figures and images for: Altered distribution, aggregation, and protease resistance of cellular prion protein following intracranial inoculation
Source: PLoS One. 2019 Jul 10;14(7):e0219457. doi: 10.1371/journal.pone.0219457 (PMC6620108; doi:10.1371/journal.pone.0219457)

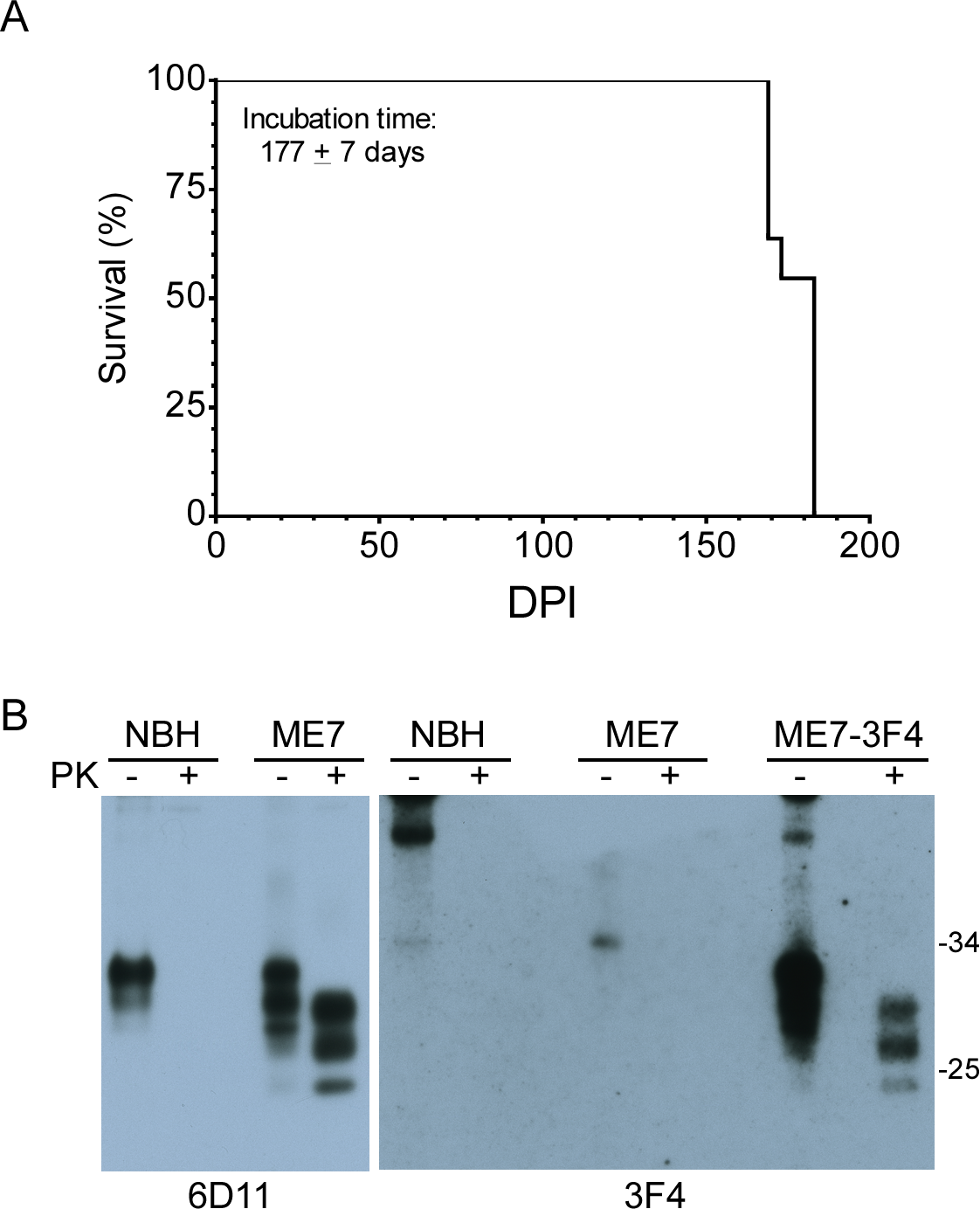

Supplement: S1 Fig — (A) Survival curve of RML mice (n = 11) inoculated IC with the ME7 strain of mouse scrapie. The average incubation time to disease in days ± SD is shown. The mice were inoculated using the same procedure and inoculum as that used for these studies. (B) Western blot analysis of PrP in normal brain homogenate (NBH) or ME7 infected brain homogenate (ME7) using the anti-PrP monoclonal antibodies 6D11 (left panel) or 3F4 (right panel). Samples were either undigested (-) or digested (+) with 63 μg/ml of proteinase K (PK). As a positive control for the 3F4 antibody, brain homogenate from an ME7 infected Tg3F4 mouse was used (ME7-3F4). Molecular mass markers are shown on the right. (TIF) [file pone.0219457.s001.tif]

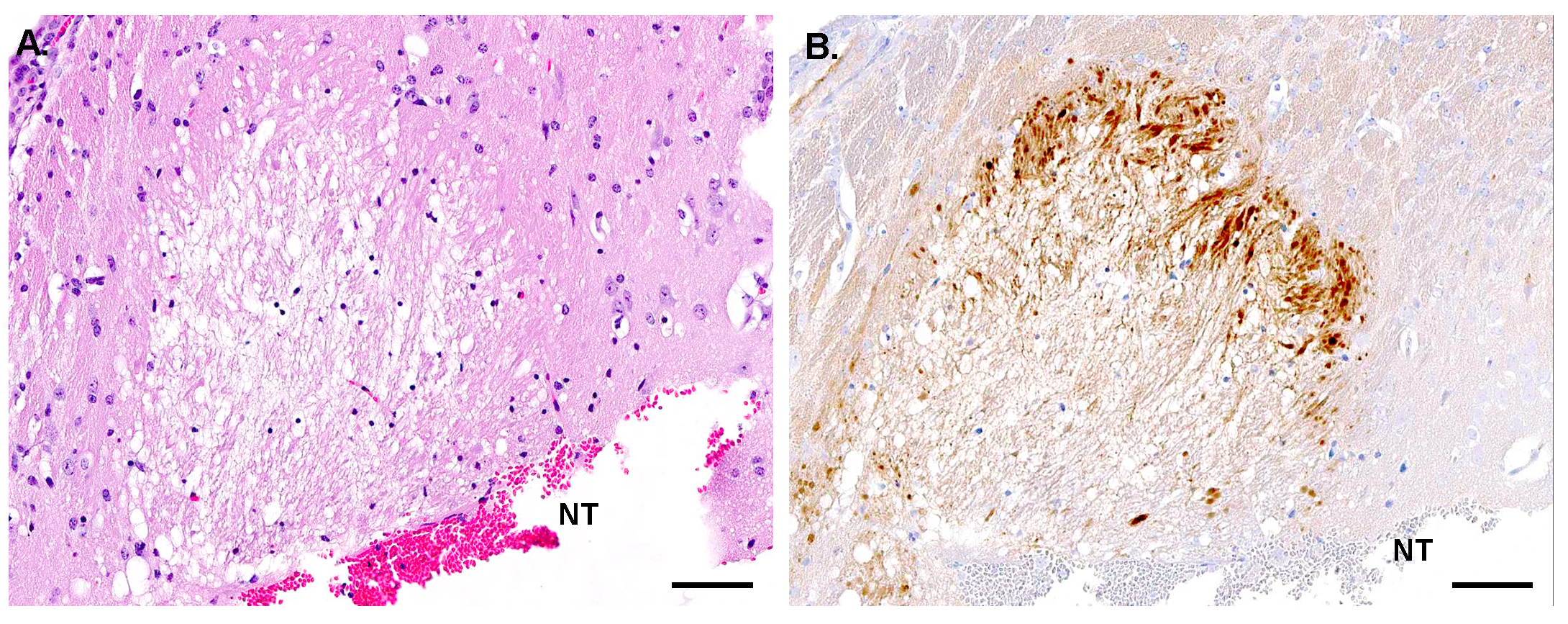

Supplement: S2 Fig — (A) H&E staining of the corpus callosum 24 hrs after stereotactic needle insertion. (B) PrP-3F4 staining in the corpus callosum 24hrs after stereotactic needle insertion. The tissue was stained using the anti-PrP mouse monoclonal antibody 3F4 conjugated to biotin as detailed in the Materials and Methods. NT = needle track. Scale bar = 50 μm. (TIF) [file pone.0219457.s002.tif]

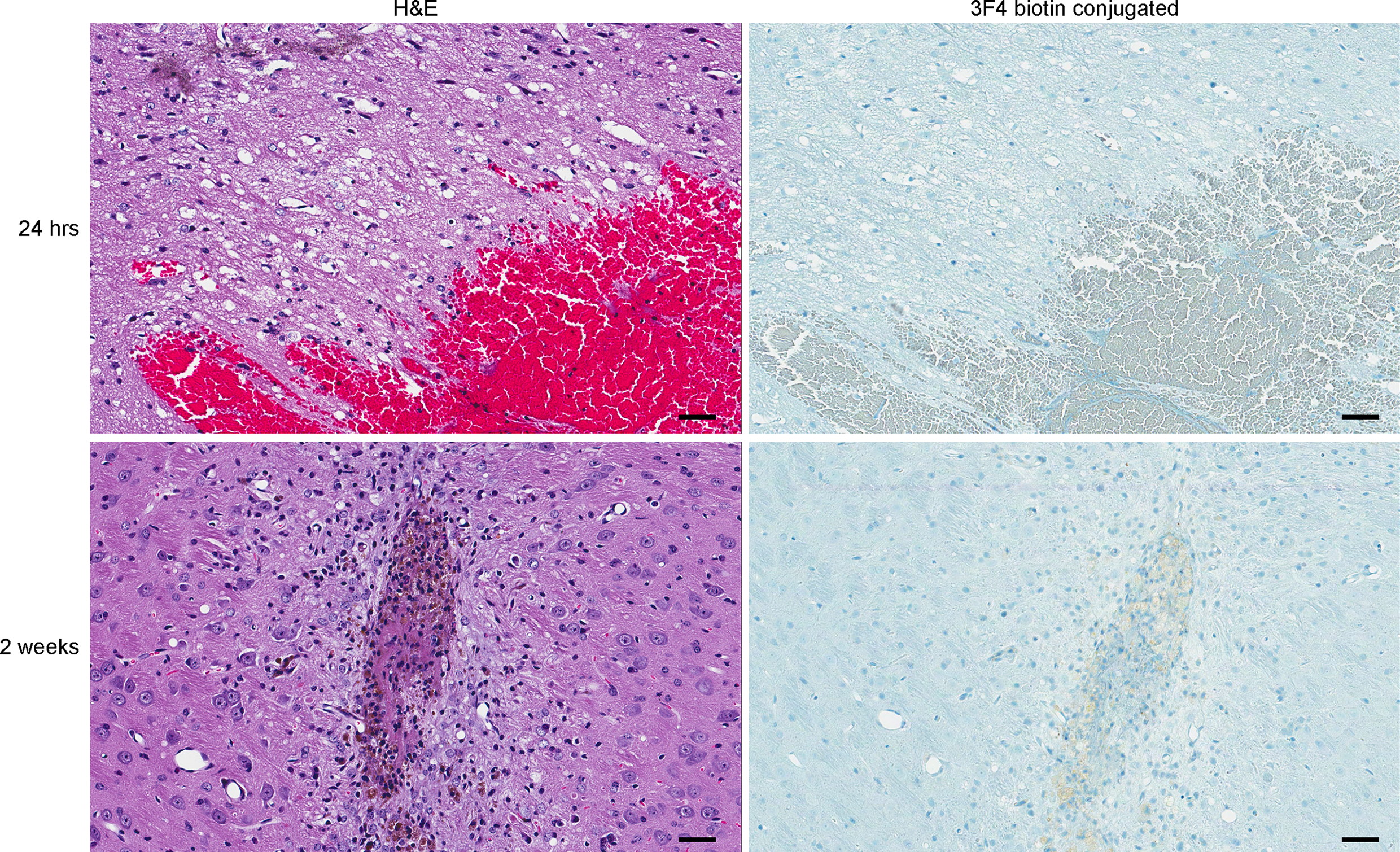

Supplement: S3 Fig — Left side panels: H&E staining of the thalamus 24 hrs and 2 weeks after inoculation with ME7 mouse prions. Right side panels: Thalamus 24 hrs and 2 weeks after inoculation with ME7 mouse prions. The tissues were stained using the anti-PrP mouse monoclonal antibody 3F4 conjugated to biotin as detailed in the Materials and Methods. Scale bar = 50 μm. (TIF) [file pone.0219457.s003.tif]

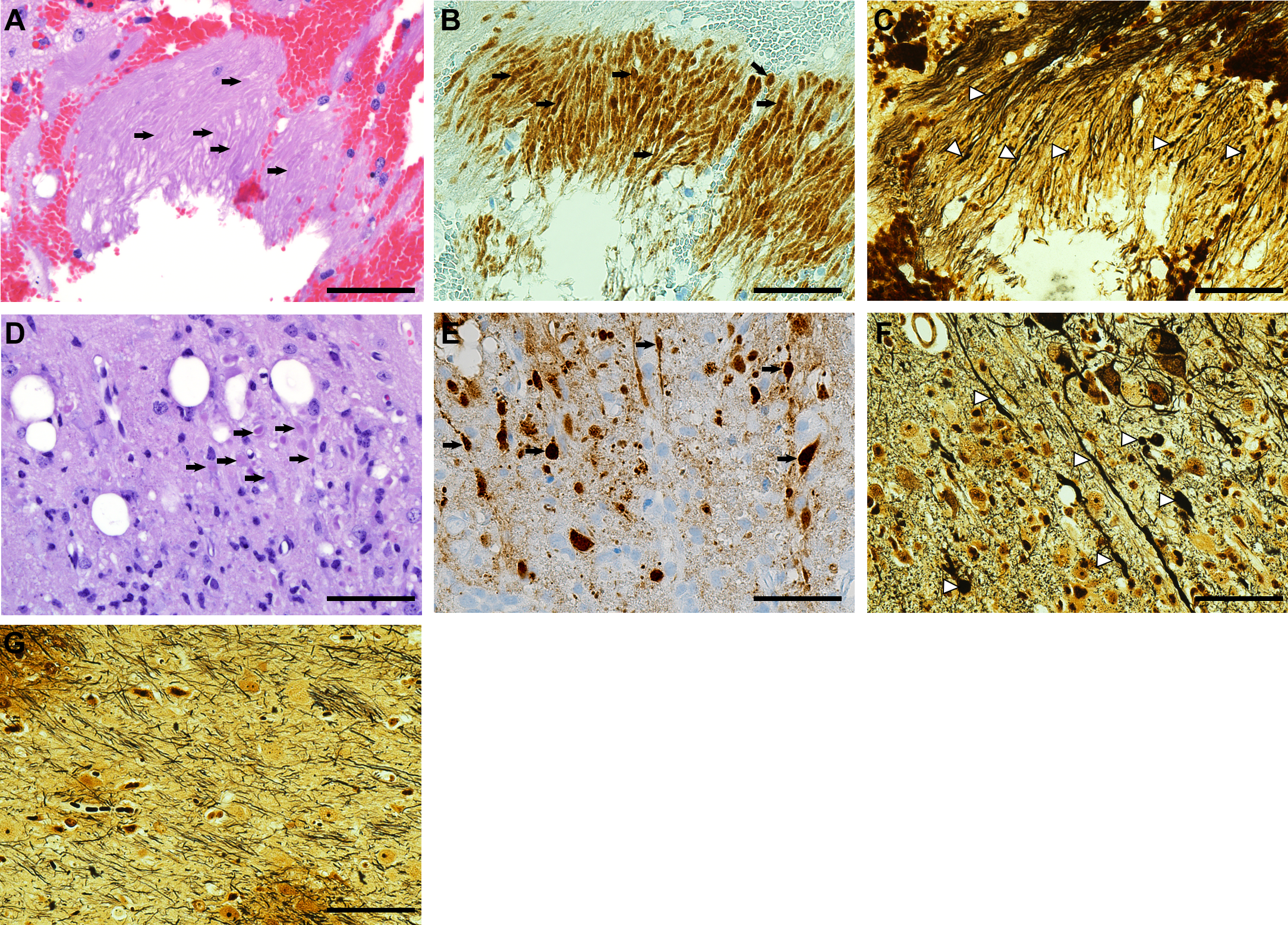

Supplement: S4 Fig — (A) H&E stain of fornix 24 hrs after inoculation of NBH. The field of view is the same as the NBH 24 hr timepoint in Fig 1B. (B) PrP-3F4 stain of fornix 24 hrs after inoculation of NBH. The field of view is the same as the NBH 24 hr timepoint in Fig 2B. (C) Bielschowsky’s silver stain for axons in fornix 24 hrs after inoculation of NBH. (D) H&E stain of thalamus one week post-inoculation with NBH. The field of view is the same as the NBH one week timepoint in Fig 1B. (E) PrP-3F4 stain of thalamus one week post-inoculation with NBH brain homogenate. The field of view is the same as the NBH one week timepoint in Fig 2B. (F) Bielschowsky’s silver stain for axons in the thalamus one week after inoculation with NBH. (G) Bielschowsky’s silver stain for axons in the thalamus of an uninoculated Tg3F4 mouse. In Panels A, B, D and E black arrows indicate examples of swollen dystrophic axons and spheroids. In Panels C and F, white arrowheads indicate examples of swollen dystrophic axons and spheroids Scale bar = 50 μm. (TIF) [file pone.0219457.s004.tif]

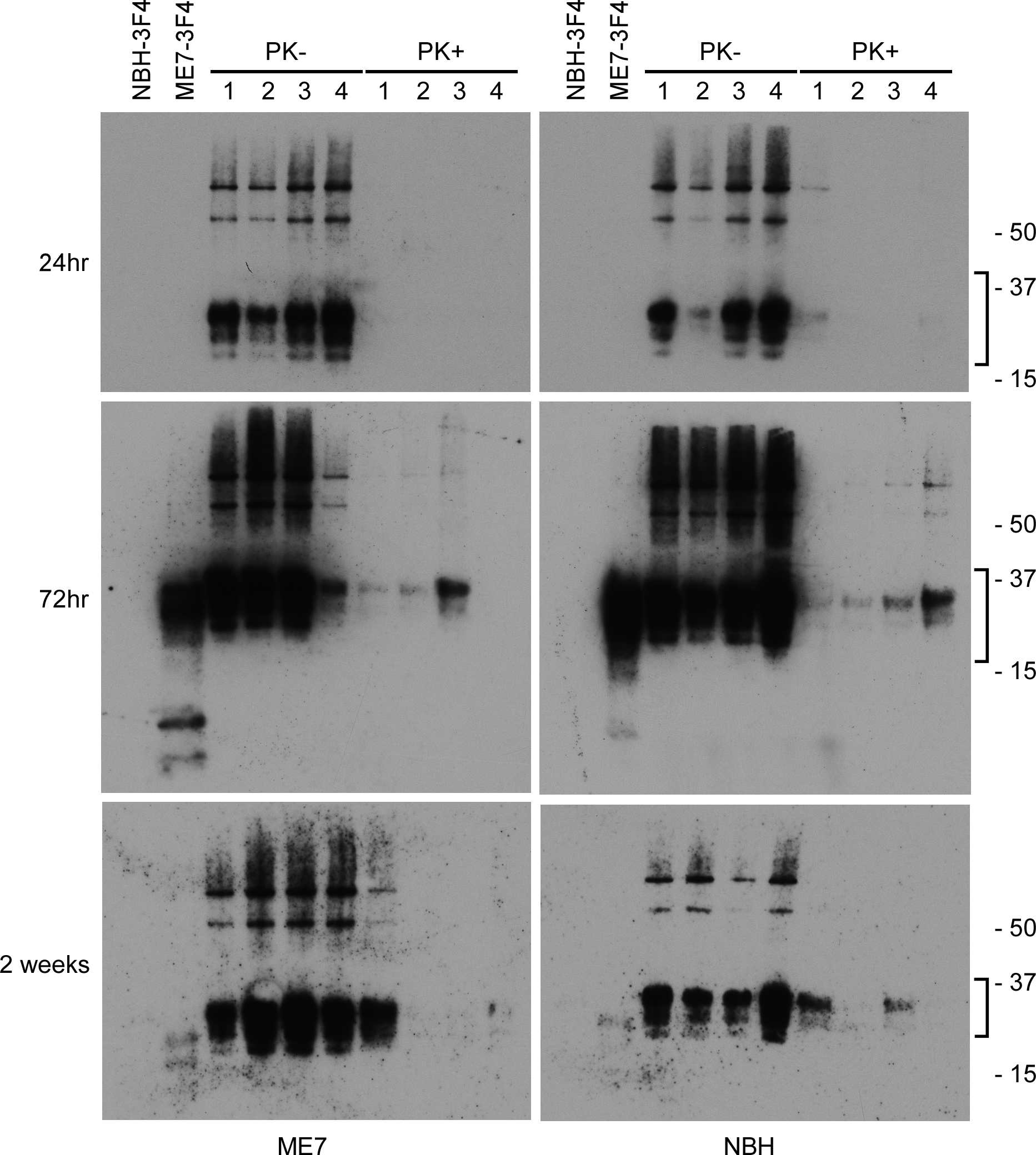

Supplement: S5 Fig — Western blots of PrP-3F4 PTA-precipitated from the ipsilateral side of the brain 24 hrs, 72 hrs, or 2 weeks after inoculation with ME7 prions or NBH. For each inoculum, tissue from 4 individual mice was assayed (lanes numbered 1–4). Lanes marked PK- show the total amount of PrP-3F4 PTA precipitated from the brain tissue. Lanes labeled PK+ show the amount of PK-resistant PrP-3F4 present in the PTA-precipitate. ME7-3F4 = PrPSc-3F4 PTA precipitated from a 1:5 dilution of ME7-3F4 prions into NBH-3F4. NBH-3F4 = PTA precipitate from an uninoculated Tg3F4 mouse brain. The bracket indicates the PrP-3F4 specific bands used for the quantitation shown in Fig 4B and 4C. Blots were developed using a biotinylated form of the anti-PrP mouse monoclonal antibody 3F4. Molecular mass markers are shown on the right side of each panel. (TIF) [file pone.0219457.s005.tif]

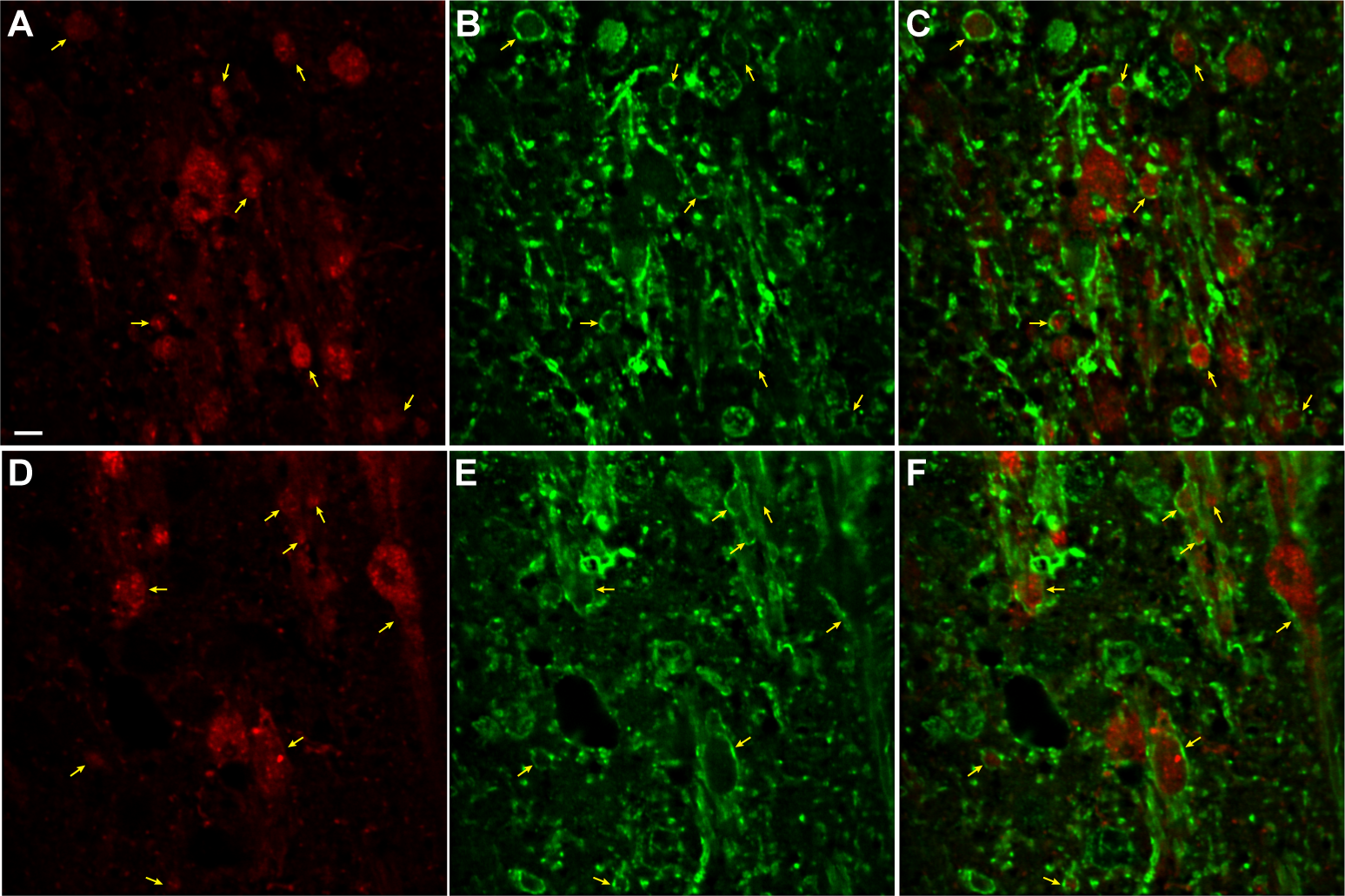

Supplement: S6 Fig — Two different fields of view from the thalamus are shown. Panels A and D show PrP-3F4 staining (red), panels B and E show PLP staining (green), and panels C and F show a merge of the two preceding panels. Yellow arrows indicate PrP-3F4 stain surrounded by PLP. The scale bar in panel A is 5 μm and applies to all panels. (TIF) [file pone.0219457.s006.tif]
