# Supplementary material for: Altered distribution, aggregation, and protease resistance of cellular prion protein following intracranial inoculation
Source: PLoS One. 2019 Jul 10;14(7):e0219457. doi: 10.1371/journal.pone.0219457 (PMC6620108; doi:10.1371/journal.pone.0219457)
Supplement: S1 Table — (DOCX) [file pone.0219457.s007.docx]

**S1 Table. Primary antibodies used for immunohistochemistry and their specificities.**

| **Target molecule** | **Cell type specificity** | **Antibody** | **IHC^1^ Dilution** | **Source** |
| --- | --- | --- | --- | --- |
| Glial fibrillary acidic protein (GFAP) | astrocytes | Rabbit polyclonal | 1:500 | Dako |
| Ionized calcium-binding adapter molecule 1 (IBA1) | microglia | Rabbit polyclonal | 1:200 | Wako |
| Myelin basic protein (MBP) | oligodendrocytes (myelin) | Rabbit polyclonal | 1:100 | Millipore |
| NeuN | neurons | Rabbit polyclonal | 1:100 | Millipore |
| Olig2 | oligodendrocytes | Rabbit polyclonal | 1:100 | Millipore |
| Prion protein | multiple | Mouse monoclonal 3F4 IgG1 biotin labeled | 1:50 | Covance |
| Proteolipid protein (PLP) | oligodendrocytes (myelin) | Rabbit polyclonal | 1:500 | ThermoScientific |

^1^ IHC = immunohistochemistry
